# Assessment of Pharmaceutical Protein–Ligand Pose and Affinity Predictions in CASP16

**DOI:** 10.1002/prot.70061

**Published:** 2025-10-04

**Authors:** Michael K. Gilson, Jerome Eberhardt, Peter Škrinjar, Janani Durairaj, Xavier Robin, Andriy Kryshtafovych

**Affiliations:** ^1^ Skaggs School of Pharmacy and Pharmaceutical Sciences, University of California San Diego La Jolla California USA; ^2^ SIB Swiss Institute of Bioinformatics Basel Switzerland; ^3^ Biozentrum, University of Basel Basel Switzerland; ^4^ Genome Center, University of California Davis California USA

**Keywords:** benchmarking, drug design, ligands, neural networks, proteins

## Abstract

The protein–ligand component of the 16th Critical Assessment of Structure Prediction (CASP16) challenged participants to predict both binding poses and affinities of small molecules to protein targets, with a focus on drug‐like compounds from pharmaceutical discovery projects. Thirty research groups submitted predictions for 229 protein–ligand pose targets and 140 affinity targets across five protein systems. Among the submitted predictions, template‐based pose‐prediction methods did particularly well, with the best groups achieving mean LDDT‐PLI values of 0.69 (scale of 0–1 with 1 best). For comparison, we also ran a set of automated baseline pose‐prediction methods, including ones using deep neural networks. Of these, AlphaFold 3 did particularly well, with a mean LDDT‐PLI of 0.8, thus outscoring the best CASP16 predictor. The CASP affinity predictions showed modest correlation with experimental data (maximum Kendall's *τ* = 0.42), well below the theoretical maximum possible given experimental uncertainty (~0.73). As seen in prior challenges, providing experimental structures did not improve affinity predictions in the second stage of the challenge, suggesting that the scoring functions used here are a key limiting factor. Overall, the accuracy achieved by CASP participants is similar to that observed in the prior Drug Design Data Resource (D3R) blinded prediction challenges. The present results highlight the progress and persistent challenges in computational protein–ligand modeling and provide valuable benchmarks for the field of computer‐aided drug design.

## 1 Introduction

When CASP was founded in the early 1990s [1], its purpose was to enable the rigorous assessment of computational methods of solving the protein folding problem; that is, of predicting the three‐dimensional structures of proteins based only upon their amino acid sequences. By forcing “blinded” predictions made without knowledge of the experimental results, CASP went beyond the prior practice, in which protein structure prediction methods were typically evaluated against protein structures that had already been published, an approach that, even when done with care, risks tilting the predictions toward the known results. Focusing primarily on the prediction of protein tertiary structure, the CASP project successfully motivated and arguably guided worldwide research and development in methods of protein structure prediction, with improving accuracy observed in the course of multiple cycles of these biennial challenges. In CASP 13 [2] (2018) and CASP 14 [3] (2020), the introduction of the AlphaFold software [4], which is based on deep neural networks (DNNs), led to step‐increases in predictive power so marked that, for the first time, one could regard the protein folding problem as having been at least partly solved. This fundamental change has freed CASP to shift its focus toward different and more complex challenges, such as predictions of protein quaternary structure and RNA structure [5, 6].

In particular, CASP 15 (2022) was the first cycle that challenged participants to predict the three‐dimensional structures of protein–small molecule (also called protein–ligand) complexes [7, 8]. Because, most drugs are small molecules that bind specific proteins, predicting protein–ligand structures is a core goal in the field of computer‐aided drug design (CADD). However, few of the ligands that appeared in the CASP 15 protein–ligand challenge were “drug‐like” compounds selected or synthesized in the context of drug discovery projects. Instead, most were naturally occurring chemical species such as enzyme cofactors (e.g., menaquinone) and metabolites (e.g., ADP), as well as metal ions (e.g., Ca^2+^). In addition, although CADD methods can provide not only the predicted conformation of a bound ligand (also known as its “pose”), but also a predicted binding affinity, affinity prediction was not part of CASP 15.

The latest round of CASP, CASP 16 (2024), also includes protein–ligand challenges, this time focused on drug‐like ligands and drawn entirely (with one exception) from industrial drug discovery projects, and it includes both poses and affinities. In addition, CASP 16 for the first time introduces a self‐assessment aspect to ligand pose predictions. The protein–ligand component of CASP 16 is very much in the spirit of prior blinded prediction challenges in the CADD space, such as CSAR [9, 10], SAMPL [11, 12, 13], and D3R [14, 15]. CASP's entry into this field is timely because these prior challenges are, at best, in abeyance, despite wide recognition of the need for ongoing CADD challenges [16].

Here, we provide an overview of the CASP 16 protein–ligand challenge component and the performance of the various participants, placing this performance into context by comparisons with baseline results from a well‐regarded docking method and newer deep learning methods, such as AlphaFold 3 [17] and Boltz‐1 [18]. Section 2 presents the molecular systems used in these challenges, the challenge procedures, and the metrics used to assess the submitted predictions. Section 3 details the submissions received, presents performance metrics, highlights high‐performing methods for poses, affinities, and reliability self‐assessments, and provides results from the baseline methods. Finally, Section 4 considers the implications of the results and places them in the context of prior pose‐ and affinity‐prediction challenges.

## 2 Methods

### 2.1 The Challenges

**FIGURE 1:**
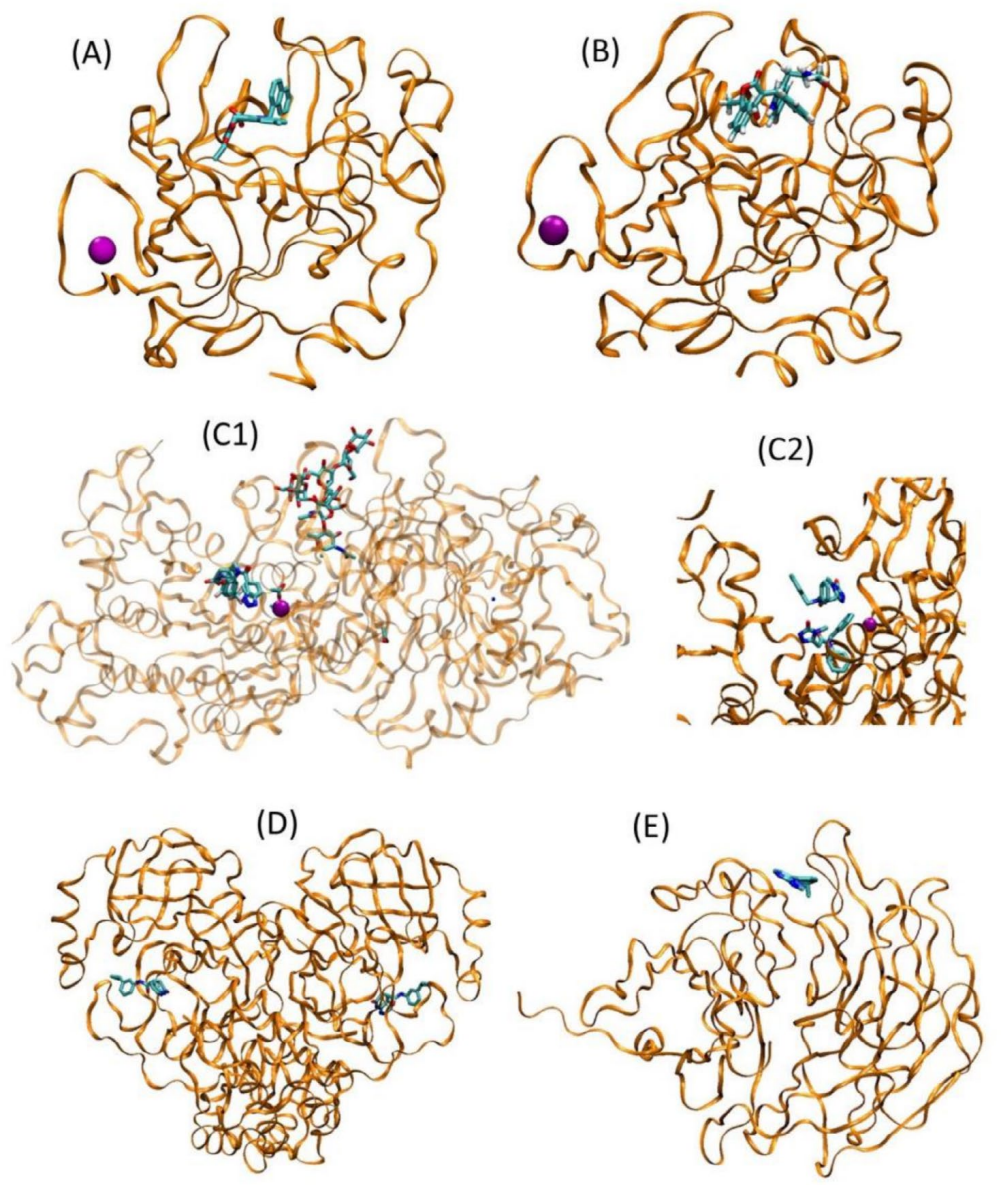
Representative crystal structures of five CASP16 pharma ligand target sets. Orange ribbons: protein backbone; purple balls: structural Zn ions; stick structures: small molecule ligands bound in the enzyme's active sites. (A) human chymase, target L1001; (B) human cathepsin, target L2001; (C) human autotaxin: (C1): Target L3053, showing Zn ion, N‐glycosylation on Asn 497, and two alternate (hence partly occupied) conformations of the bound ligand. (C2): Close‐up of the binding site of target L3018, showing two fully occupied instances of the same ligand, one deeper in the binding site and the other bound on top of the first instance; (D) SARS‐CoV‐2 main protease (Mpro) with bound ligands, target L4020; (E) human protein WDR55 with bound ligand, target L5001.

The present challenges were based on protein–ligand datasets for five proteins, as listed in Table 1 and Table S1, depicted in Figure 1, and summarized in this section. Further details of these molecular systems and the experiments used to generate the data are provided in the companion paper from the contributing groups at Hoffmann‐La Roche (L1000–L3000) and Idorsia Pharmaceuticals (L4000) [19], and in a separate paper from the Structural Genomics Consortium (L5001) [20]. In some cases, both the structure and affinity of the protein–ligand pair had been experimentally determined and were therefore available as prediction targets. In others, only the structure or only the affinity was available.

**TABLE 1:** Protein–ligand systems used in CASP16 challenge.

Protein	*N*_pose_	Xtal Resol (Å)	*N*_affinity_	Affinity range	Contributor	Supertarget	Targets
Chymase	17	1.1–2.2 Mean 1.7	Stage 1: 17 Stage 2: 17	1–400 nM	F. Hoffmann‐La Roche	L1000	L1001–L1017
Cathepsin G	2	1.2, 1.2	0	NA	F. Hoffmann‐La Roche	L2000	L2001, L2002
Autotaxin	189	1.3–2.7 Mean 1.9	Stage 1: 123 Stage 2: 93	1 nM–10 μM	F. Hoffmann‐La Roche	L3000	L3001–L3231
Mpro	20	1.3–1.9 Mean 1.7	0	NA	Idorsia Pharmaceuticals Ltd.	L4000	L4001–L4028
WDR55	1	1.2, 1.2	0	NA	Structural Genomics Consortium		L5001

Abbreviations: *N*_affinity_, number of affinities to be predicted; *N*_pose_, number of ligand poses to be predicted; Supertarget, CASP ID number for the collection of data associated with this protein (WDR55 is not considered a supertarget because it corresponds to only one prediction target); Xtal Resol, range of crystallographic resolutions of the cocrystal structures.

In some cases, the crystal structure included not only the drug‐like ligand of interest but also non‐drug‐like ligands from the crystallization liquid. These so‐called incidental ligands comprise sulfate, acetate, bromide, and chloride ions; dimethyl sulfoxide; ethylene glycol; and tetraethylene glycol. We identified the incidental ligands with a heavy atom ≤ 4.5 Å from any heavy atom of the drug‐like ligand and invited participants to include these in their structural predictions. However, we treated these predictions separately and regarded them as being of lower importance, for two reasons. First, such predictions are not representative of the main application of the prediction methods, namely computer‐aided drug discovery. Second, the incidental ligands probably bind with relatively low affinity and appear in the crystal structures only because they are present at rather high concentrations in the crystallographic liquor, so failure to predict the structure of such weak‐binding ligands is not considered a major weakness of a computational method.

Chymase is a globular, 247‐residue enzyme with a structural Zn outside the active site (Figure 1A). The challenge dataset contains 17 individual chymase–ligand targets with both structures and affinities to be predicted.

Cathepsin G is a 255‐residue globular enzyme, structurally similar to chymase (Figure 1B). The prediction dataset contains two protein–ligand structures; no affinities were to be predicted.

Autotaxin is a larger (846‐residue), single‐chain, globular enzyme, with one or two Zn ions in or near the active site (depending on the structure), a posttranslational N‐glycosylation far from the active site (Figure 1C1) and incidental ligands in some of the crystal structures. The autotaxin dataset comprises 93 protein–ligand pairs with both structures and affinities to predict; 96 with only structures to predict; and 30 with only affinities to predict. The protein sequence is a hybrid construct, with most of the structure derived from rat, which is easier to crystallize, but with binding site residues from the human protein. The construct also includes a His tag. Some crystal structures provided two alternate (hence partly occupied) ligand conformations (e.g., Figure 1C1, whereas others included two fully occupied ligand conformations Figure 1C2). In such cases, we scored the agreement of the predicted ligand poses against all available conformations in the crystal structure and used the best score in computing performance statistics.

The SARS‐CoV‐2 main protease, MPro, is a medium‐sized, homodimeric globular enzyme with 306 residues per chain. There were originally 25 protein–ligand structures (but no affinities) to predict, but we report statistics only on 20. This is because we were informed on February 26, 2025, that the following five MPro structure targets had become available in the PDB well before the CASP 16 submission deadline: L4006 (PDB ID 7gs2), 4007 (7grr), 4008 (7grw), 4009 (7grh), and 4010 (7gri). The statistics presented in this paper have been recomputed, relative to those presented at the CASP16 conference, with these targets omitted. Because, there are two chemically equivalent binding sites, the crystal structures typically included a separate copy of the ligand in each binding site (Figure 1D). Submitted predictions might include one ligand in one of the binding sites or two ligands, one in each binding site. We scored the agreement of all available predicted ligand poses against all available conformations in the crystal structure, accounting for symmetry, and used the single best score in computing performance statistics. Some ligands are covalently bound to the sulfur atom of Cys. The attachment points for each covalent inhibitor are defined by the first atom of the following SMARTS strings: L4003, [cX3][nX2][cX3][nX2]; L4013, [C](=[N])[c]; L4019, [C](=[N])[c]; L4023, [C](=[N])[c]. Some of the MPro structures also include incidental ligands.

The final target is human protein WDR55, a 383‐residue WD repeat protein, which is not an enzyme and also is not so far considered a drug target. Its structure was solved with a single bound ligand (Figure 1E). Unlike the other target proteins considered above, WDR55 did not have a known, well‐characterized binding site. Moreover, whereas the other ligand–protein complexes used in this challenge had maximum similarity scores (of range 0–1) to an existing structure in the PDB ranging between 0.3 and 0.9, the similarity score for the WDR55 target was 0.0 (see Section 2.5 for further details).

### 2.2 Structure of the Challenge

Detailed information and submission instructions were provided via the CASP website (https://predictioncenter.org/casp16). Structure predictions were required to include three‐dimensional coordinates of the protein in PDB format and three‐dimensional coordinates of the ligand in MDL molfile format in order to avoid ambiguity regarding atom identities and connectivity. The website provided the following information about each protein–ligand target: a brief description of the dataset; protein sequence; ligand SMILES string with any available stereochemical specifications; a statement of what was to be predicted, that is, structure only, affinity only, or both structure and affinity; and, for structure predictions, a template protein PDB file with the coordinate fields filled with zeroes as an unambiguous guide to what was to be predicted. Affinity predictions were accepted in the form of “absolute” affinities (i.e., dissociation constants *K*_*d*_), “relative affinities” (i.e., the ratio of each ligand's dissociation constant to that of an arbitrarily selected reference ligand); or ligand rankings within each supertarget dataset. For each pose‐ or affinity‐prediction target, participants were permitted to submit up to five different predictions, termed Model 1 to Model 5, with the understanding that Model 1 would be considered their best or most favored prediction. Participants were also asked to submit text describing the methods they used to make their predictions.

The information needed for supertargets L1000–L4000 (chymase, cathepsin G, autotaxin, and Mpro) was posted May 5, 2024, and predictions were due by July 21, 2024. After that, the files submitted by participants were checked for technical validity (e.g., use of the correct PDB structure template and correspondence of the ligand MDL format to the provided SMILES string), and the participants were notified of any apparent problems and allowed to submit corrected files by August 4. After this, we proceeded to the next prediction stage, where we released experimental coordinates of protein–ligand complexes and asked modelers to predict binding affinities for each of them; note that in the first stage, the task was to predict affinities without the benefit of the experimental structures. Stage 2 ran from August 7 to August 21. The last ligand target, L5001, WDR55, arrived at CASP later and was shared with participants on August 9, 2024, with all predictions due by August 28, 2024. In the process, we discovered that there was an inaccuracy in the SMILES record for this target and re‐released it as L5001v1 on August 30, with the submission deadline of September 18, 2024.

Participants were also invited to submit estimates of the reliability of each of their structural predictions. This is expressed as a number, called the LScore, in the range [0, 1], where 0 means low reliability, and 1 means high reliability.

By default, CASP expects one laboratory to test one method. However, sometimes a lab wishes to test more than one method, and CASP provides a mechanism for this through multiple group registrations from the same lab (see predictioncenter.org/casp16/registration.cgi). Thus, it should be understood that more than one “group” may be associated with a given lab. Each group has a unique number, for example, group 494, which was automatically assigned for use during the anonymized assessment process, and a unique name, such as ClusPro, given by the participant, which was revealed to the assessors only following the initial assessment.

### 2.3 Evaluation Metrics

As detailed in Sections 3.1 and 3.2, participants were invited to submit predictions of protein–ligand binding affinities and of the structures of protein–ligand complexes. Our assessment of predicted protein–ligand structures focused on the accuracy of the ligand poses, that is, of their locations and conformations, and of the binding site structures. We did not assess the accuracy of the overall protein structures because multiple crystal structures of these target proteins were already available in the PDB. This section describes the basic metrics used to assess accuracy, while the details of how these were used to compare accuracy across groups are presented in the respective subsections of Results.

We evaluated pose accuracy in terms of two metrics. One pose‐accuracy metric is the binding‐site superposed, symmetry‐corrected, pose root‐mean‐square deviation (BiSyRMSD, here further abbreviated as RMSD) [8]. This is computed by defining binding site residues as any residue with heavy (non‐hydrogen) atoms within 4 Å of heavy atoms of the ligand, superimposing Cα atoms of those binding site residues of the predicted and crystallographic protein–ligand structures with the Kabsch algorithm [21], and then computing the root‐mean‐square distance between the predicted and crystallographic coordinates of corresponding ligand atoms. In case of symmetries, such as the 180° rotation of a phenyl group, we take the lowest root‐mean‐square distance achievable by the product of all symmetry operations. The best possible RMSD is 0, and there is no mathematical upper limit. The second pose‐accuracy metric is an enhanced version of the previously described local distance difference test for protein–ligand interactions (LDDT‐PLI) score [8, 22]. The LDDT‐PLI is obtained by computing the differences of interatomic distances between ligand atoms and binding site atoms in the crystal structure and the predicted complex. In contrast to CASP15, an additional penalty is applied to predicted ligand–protein contacts that are not present in the crystal structure [22]. The LDDT‐PLI favorably scores the correct prediction of close ligand–protein contacts and ranges from 0 (no crystallographic contacts predicted correctly) to 1 (all crystallographic contacts predicted correctly). The accuracy of each predicted binding site structure was evaluated in terms of the root‐mean‐square deviation of Cα atoms in the binding site (BB‐RMSD [8]) and the differences in interatomic distances of all binding site atoms (LDDT‐LP [8], for “ligand pocket”). When crystallographic or predicted structures contained multiple protein chains or multiple copies of identical ligands, all permutations were scored, and we focused on the permutation with the best LDDT‐PLI score. BB‐RMSD and LDDT‐LP are reported based on the best ligand RMSD. All accuracy scores were computed with OpenStructure [22] version 2.8.

We evaluated the affinity prediction accuracy in terms of ranking power, as measured by Kendall's *τ* statistic [23], because this allows all submissions—whether provided in terms of absolute, relative, or ranked affinities—to be compared against each other. In order to put the participants' results into context, we also estimated the expected best achievable value of Kendall's *τ* for each dataset. The idea here is that even a computational method that yields perfect predictions will not give a perfect correlation with experiment because the experimental data are subject to error. We used a common estimate that the experimental IC50 data could be off by about a factor of 3, which corresponds to a binding free energy of DG = RT ln (3) = 0.66 kcal/mol at room temperature. For each affinity target (chymase and autotaxin), we converted the experimental IC50 data into estimated binding free energies ΔG≈RTlnIC50 and used resampling to generate 1000 new datasets with added experimental error drawn from a Gaussian distribution with a mean of 0 and a standard deviation of 0.66 kcal/mol. We computed Kendall's *τ* for each resampled data set against the actual experimental data set and determined its mean and standard deviation. The mean represents the highest value of Kendall's *τ* that even a perfectly accurate computational method is expected to achieve. We also repeated this exercise with a standard deviation of 2 kcal/mol, which corresponds with a factor of about 30 uncertainty in IC50, in order to look at how the mean resampled Kendall's *τ* degrades with increased assumed experimental error.

The reliability estimates (LScores, Section 3.3) were evaluated in terms of the correlation between the LScore and the LDDT‐PLI of each structural prediction. Thus, we hope to see greater accuracy for predictions considered more reliable. The correlation between LScore and LDDT‐PLI across structure predictions was summarized with Kendall's *τ* ranking statistic.

### 2.4 Baseline Predictors

#### 2.4.1 Pose Prediction

Results from CASP participants were compared to those we generated using the following “baseline” predictors: three DNN methods, AlphaFold 3 (AF3), Boltz‐1 (v0.4.1), RoseTTAFold All‐Atom (RFAA) [24]; and AutoDock Vina (AD Vina) [25, 26], a well‐regarded docking method used in conjunction with SWISS‐MODEL [27]. For AlphaFold 3, we used the average per‐atom pLDDT of the ligand, as provided by AlphaFold 3, as a proxy for LScore. Note that AlphaFold 3 and Boltz‐1 were not publicly available in a form suitable for protein–ligand pose predictions during the actual challenge, so these methods could not be used by CASP16 participants. Pose predictions from each baseline predictor were scored as described in Section 2.3.

The baseline methods AF3 and Boltz‐1 were run on one A100 40 GPU node each with 10 recycling steps, 200 diffusion sampling steps, 5 seeds, and 5 diffusion samples per seed, which resulted in a total of 25 models per system for each method. To ensure that the DNN methods were compared on an equal footing, we used the same MSAs as input for both AF3 and Boltz‐1. The standard AF3 MSA generation pipeline was run to obtain the non‐paired and paired MSAs for each system. The same pairing keys were used to generate the custom MSA CSV files for Boltz‐1. For unpaired sequences, pairing keys were set to −1. For RFAA, we followed the standard procedure with a number of MAXCYCLE set to 10.

For the docking protocol, we docked all target ligands using AD Vina within one single bound conformation of each protein receptor. The template for the protein receptor was obtained through SWISS‐MODEL [28] for each supertarget. In brief, we used the reference FASTA sequence for each supertarget as input, which serves as a query to evolutionarily related protein structures against the SWISS‐MODEL Template Library (SMTL) [27]. The best template was selected based on the sequence identity to the reference sequence and the presence of a ligand in the main known pocket, regardless of its nature. The following templates were chosen: 5yjp [29] for chymase (L1000), 1cgh [30] for cathepsin G (L2000), 5s9m [31] for autotaxin (L3000), and 7n8c [32] for Mpro (L4000). Once a template was selected, a 3D protein model was automatically generated by first transferring conserved atom coordinates as defined by the target‐template alignment. If necessary, residue coordinates corresponding to insertions/deletions in the alignment were generated by loop modeling, and a full‐atom protein model was obtained by constructing the nonconserved amino acid side chains. The template structure was then prepared by adding hydrogen atoms using REDUCE [33] and then converting to PDBQT file format using Meeko [34]. Ligands were prepared using Scrubber [35] to adjust the protonation state at pH 7.4 and converted to PDBQT using Meeko. The center of the box was defined based on the ligand present in the template, and the search box dimensions were set to 25 × 25 × 25 Å. AD Vina was then used to dock each ligand, except for the four covalent MPro ligands, using the default parameters; in particular, exhaustiveness was set to 8. The best pose was selected based on the docking score.

#### 2.4.2 Binding Affinity Prediction

Binding affinity predictions for Stage 1 from the CASP participants were compared to two commonly used baseline predictors: molecular weight (MolW) and the partition coefficient *P* (Log*P*), both calculated using RDKit [36]. We also evaluated performance against a classical regression model, the Gaussian process regressor (GPR) [37] in combination with a Tanimoto similarity kernel, fitted on publicly available ChEMBL data (accessed on December 5, 2024) [38]. A separate GPR model was trained for each supertarget, chymase (L1000) and autotaxin (L3000), using the CHEMBL3691 and CHEMBL4068 datasets, respectively. These datasets contained 1110 IC50 values for human chymase, ranging from 0.3 nM to 570 μM, and 450 IC50 values for human autotaxin, ranging from 10 pM to 1.25 mM. Molecule SMILES from both datasets were featurized using the Morgan fingerprint method from RDKit, with chirality information included (includeChirality = True). Additionally, we used the AD Vina scores from the calculations above as an additional affinity predictor.

For Stage 2, we used the scoring functions from GNINA [39, 40] (v1.3) and AD Vina as baseline predictors for binding affinities, leveraging the ground truth protein–ligand complexes. Following the same preparation protocol described earlier for the naïve docking protocol, protein structures and ligands were converted to PDBQT format and provided as inputs to GNINA and AD Vina. The ligands were then locally minimized, and the resulting docking scores were used as baseline predictions for binding affinities in Stage 2.

### 2.5 Assessing the Existence of Similar Protein–Ligand Structures in the PDB

To assess whether the availability of similar protein structures in the PDB affected the performance of the prediction methods, we used Foldseek [41] to identify structures with the same or similar fold for each protein in the CASP16 protein–ligand challenge. For each protein–ligand target, we quantified ligand similarity by calculating the Combined Overlap Score (SuCOS) [42] between the molecule in the CASP target and the corresponding molecule(s) in the template. In brief, the SuCOS quantifies the degree of volume overlap and the overlap of chemical features such as hydrogen bond donors and acceptors between a query molecule (here CASP targets) and a reference molecule (here a template molecule). The SuCOS ranges from 0, where there is no overlap of the volume and chemical features onto the template molecule, to 1, where there is a perfect overlap. The alignment between a target molecule and a template molecule was obtained using *rdShapeAlign* from RDKit (v2024.09.6), and the SuCOS was obtained using the implementation from https://github.com/susanhleung/SuCOS. Additionally, we measured the pocket coverage by comparing the sequence overlap between the ligand‐binding pockets of the target and the template using the sequence alignment from Foldseek. Overlap was defined as the number of residues in common between the two binding pockets normalized by the number of residues in the target pocket. Residues belonging to a pocket were solely identified based on their distance from the ligand using a cutoff of 6 Å. Note that the definition of binding site is loosened for similarity calculations, as, unlike in accuracy assessment, the query and target no longer necessarily have the same side chains. Finally, the similarity score was obtained by multiplying the SuCOS by the pocket coverage, and we used the highest score across templates as a measure of how closely the structural and ligand‐binding features of each CASP target were represented in the PDB. Further details on the template search protocol can be found in Škrinjar et al. [43]. The template cutoff dates were set to the training cutoff dates used for each DNN method; September 30, 2021, was used for both AF3 and Boltz‐1, whereas March 31, 2020, was used for RFAA. For CASP participants and the naïve docking protocol, we used May 3, 2024, which corresponds to the deposition date of the supertargets L1000, L2000, L3000, and L5000 datasets.

## 3 Results

This section compares the accuracy of pose predictions of drug‐like (Section 3.1) and incidental ligands (Section 3.2); affinity predictions (Section 3.3); and reliability self‐assessments (Section 3.4) across groups. We also consider potential correlates of accuracy, such as ligand complexity (Section 3.1.3), and examine the accuracy of several DNN–and docking‐based pose‐prediction methods (Section 3.1.4).

### 3.1 Pose Predictions for Drug‐Like Ligands

#### 3.1.1 Submissions

Thirty research labs participated in the CASP16 protein–ligand challenge, with 34 CASP pose‐prediction groups for the pharmaceutical ligands and 19 for the incidental ligands. (As noted above, one lab can submit as more than one “group”.). In Stage 1, there were 28 and 21 groups for the chymase and autotaxin affinity targets, respectively, and the corresponding numbers for Stage 2 are 27 and 24. Only 13 groups provided LScores with their pose predictions. Of these, three groups submitted only LScore values of 1, and one group submitted negative (hence out of range) LScore values, so we analyzed only the remaining 10 submissions. Although most groups making Stage 1 affinity predictions also made Stage 2 affinity predictions, some groups submitted affinity predictions for only Stage 1 or only Stage 2. The methods used by participants included physics‐based modeling (though none used the highly regarded approach of simulation‐based free energy calculations [44, 45]), methods based on artificial intelligence with DNNs (AI), and template‐based methods, which predict new structures based on similar structures available in the PDB. Many groups combined several techniques, such as a combination of different AI methods or a workflow that uses a structural template if available but falls back on docking calculations if no template can be found. Many methods combined the participants' in‐house software with open‐source and/or commercial software, with different codes carrying out different tasks in a computational pipeline. One distinctive submission relied on citizen‐scientist docking with the GUI‐driven DockIt code. Participants were asked to state the availability of their respective methods in their abstracts, which are available on the CASP prediction center website: https://predictioncenter.org/casp16/doc/CASP16_Abstracts.pdf.

#### 3.1.2 Assessments

**FIGURE 2:**
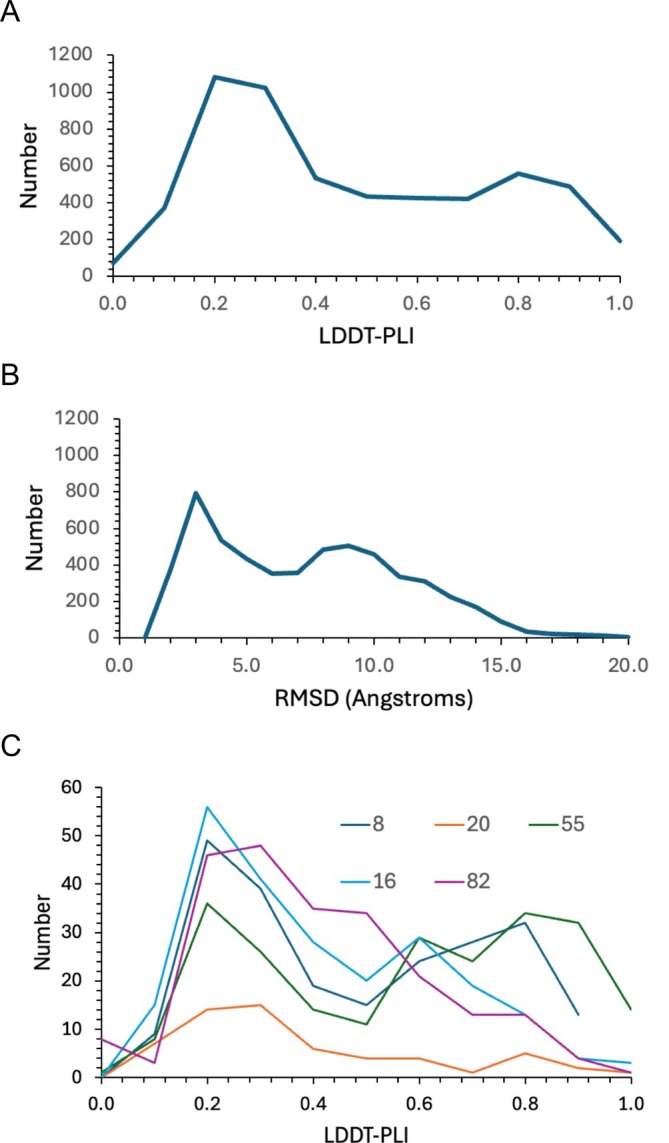
Pose accuracy statistics across all groups. (A) Histogram of LDDT‐PLI values (bin width 0.1). (B) Histogram of RMSD (Angstroms; bin width 1.0). (C) Histograms of LDDT‐PLI values for five arbitrarily selected groups.

Histograms of pose prediction accuracy across all submissions are bimodal, whether evaluated in terms of the LDDT‐PLI (Figure 2A) or RMSD (Figure 2B). In many cases, this bimodal distribution is observed for individual groups (Figure 2C).

We also computed a metric of the accuracy of predicted structures of the binding site itself, BB‐RMSD (Section 2.4), but ultimately chose not to rank participants on this metric. This is because predictions with accurate ligand poses (i.e., low RMSD or high LDDT‐PLI) also tend to have accurate binding site predictions (BB‐RMSD). In particular, if ligand RMSD < *X* Å, then typically BB‐RMSD < *X* also (Figure S1).

Most groups included predictions for all or nearly all of the 229 pose prediction targets, but some included a more modest number of predictions (Figure S2A). We thus had to consider whether and, if so, how to penalize omitted predictions. As shown in Figure S2B, the distribution of mean accuracy across targets is about the same for groups that submitted few versus many predictions (left vs. right batch of points). Although the two points with the highest mean LDDT‐PLI in the left batch (red circle) are somewhat above the best mean LDDT‐PLI values to the right of the graph (green circle), these particularly high‐quality predictions are mainly for chymase, which was also very well predicted by the groups that modeled all targets (data not shown). We therefore chose to favor groups that included predictions across most or all targets and penalize those that skipped targets. To do this, we compute the skip‐penalized mean LDDT‐PLI of a given group as the mean of its LDDT‐PLI values when skipped predictions are assigned an LDDT‐PLI value of zero. In order to provide a metric that might be more familiar to CADD developers and users, we also defined a pose prediction as successful if RMSD ≤ 2.5 Å [46, 47, 48, 49, 50] (see sample structures with RMSD = 2.5 Å in Figure S3) and computed the skip‐penalized success rate of a given submission by treating skipped targets as unsuccessful predictions. As shown in Figure S4, for all but the least accurate groups (low success rate, low LDDT‐PLI), the skip‐penalized success rate and the LDDT‐PLI score correlate extremely well.

**FIGURE 3:**
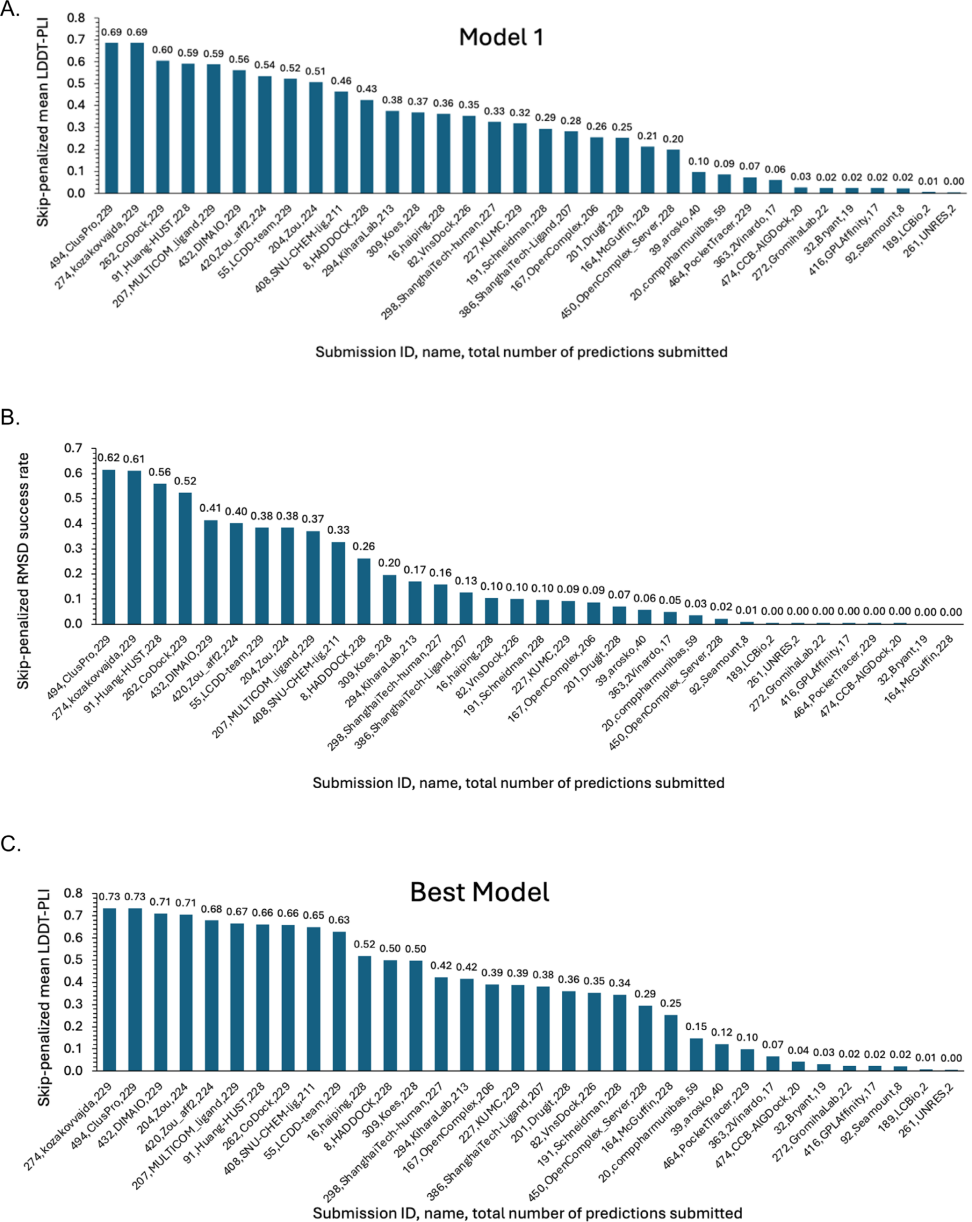
Accuracy rankings by group of pharmaceutical ligand pose predictions, based on skip‐penalized mean LDDT‐PLI for Model 1 (A); skip‐penalized RMSD success rate for Model 1 (B); and skip‐penalized mean LDDT‐PLI for the best model submitted for each target (C). Each column is one group and is labeled as group ID, method name, and number of predictions submitted.

Thus, as shown in Figure 3, both metrics provide very similar rankings of the overall performance of the various submissions, with leading accuracy from groups Cluspro (494), kozakovvajda (274), CoDock (262), Huang‐HUST (91), and several others. (The LDDT‐PLI statistics for each separate Supertarget (chymase, cathepsin G, autotaxin, Mpro, and WDR55) are provided in Table S2.) Importantly, groups that generated more accurate submissions on one supertarget tended to generate more accurate predictions on other supertargets also, with *R* ^2^ values of 0.46, 0.53, and 0.61 (Figure S4), so there is a degree of consistency across cases. All four groups include template‐based steps. The leading ClusPro and kozakovvajda groups used the same methodology run with different parameters (Kozakov, pers. commun.). The citizen‐scientist Drugit group did not rank among the most performant methods.

**FIGURE 4:**
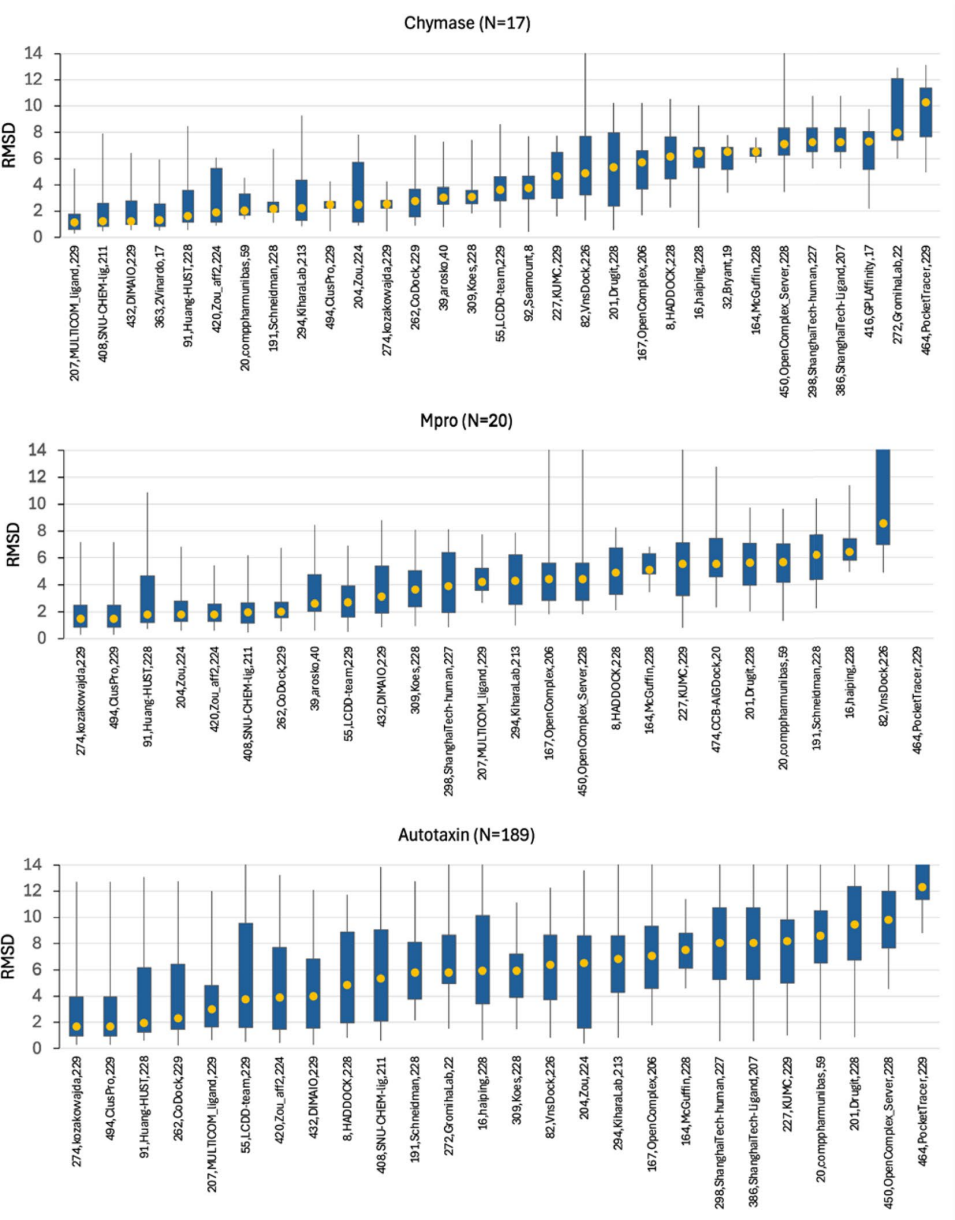
Pose RMSD distributions by group for each supertarget with more than two targets. The vertical axis limit is set to 14 to focus on the lower‐RMSD results and for comparison with prior challenges [14, 15] which used similar axis limits. Skinny lines indicate minimum and maximum, solid boxes indicate first and third quartiles, and orange dots indicate medians. No penalty was applied for skipped predictions.

It is also of interest to compare the overall accuracy of the CASP16 pose predictions with that observed in the prior D3R challenges. Figure 4 facilitates this comparison by plotting RMSD statistics in the D3R format; these plots should be compared with Figure 1A in the Grand Challenge 2 paper [51], Figure 2A in the Grand Challenge 3 paper [15], and Figure 3A in the Grand Challenge 4 paper [14]. Due to the large variation across protein targets, here and in D3R, as well as the modest number of targets, we find it difficult to conclude that the present results are better or worse than the prior ones.

Having focused so far on the Model 1 predictions submitted by each group, we now consider performance across the maximum five models submitted by each group. To do this, we compare the mean skip‐penalized LDDT‐PLI statistic of each group's Model 1 submission with the corresponding mean of their best (highest LDDT‐PLI) prediction for each target. (If a group submitted only a Model 1 prediction, this was also their best prediction.) We find that the mean skip‐penalized LDDT‐PLI across all groups rises from 0.30 to 0.37 on going from Model 1 to the best of Models 1–5, and the two means are highly correlated, as shown in Figure S6. Thus, in general, the best model for a given target tended to be only a little more accurate than Model 1. Still, considering the best model leads to some reranking of the submissions: Although the two top‐ranked groups based on Model 1, Cluspro and kozakovvajda, are also top‐ranked when considering the best across all models, there is some shuffling of the subsequent rankings (Figure 3C vs. Figure 3A).

**FIGURE 5:**
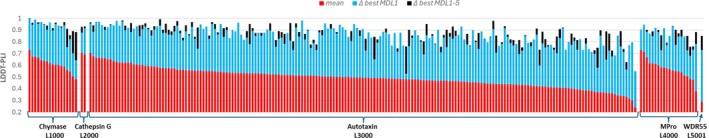
Accuracy of predictions sorted, for each supertarget, in descending order of the mean of all groups' best LDDT‐PLI across all models for each target. One bar represents one target. Red: per‐target mean across groups' of each group's best LDDT‐PLI score. Blue: increment between the mean shown in red and the maximum Model 1 LDDT‐PLI across groups. Height of the red + blue bars indicates LDDT‐PLI of the best Model 1 across groups. Black: Increment between the maximum shown in blue and the maximum LDDT‐PLI across all models of all groups. Absence of a black bar indicates that the highest‐scoring model was Model 1.

We looked for patterns in the “predictability” of individual targets (ligand‐protein pairs) across groups. As shown in Figure 5, average performance for chymase, cathepsin G, and Mpro tended to be somewhat more accurate than that for the other two targets, with accuracies (measured as mean best non‐skip‐penalized LDDT‐PLI) of 0.62, 0.49, and 0.57, respectively (red bars). This pattern is also evident when analyzing per‐group performance in terms of RMSD score (Figure 4). The average performance on WDR55 is among the worst, presumably because of the novelty of this system (Section 2.1). Although the four covalent Mpro ligands might seem to offer a special challenge to pose‐prediction methods, their mean scores are unremarkable, at 0.48, 0.51, 0.51, and 0.58, respectively.

Even though an “average” CASP16 group scored above LDDT‐PLI of 0.6 only on 17% of targets (28/229 red bars), there were very good models on practically all the targets, as the LDDT‐PLI of the best submitted model exceeded 0.6 on 98% of targets (224/229 whole bars), including 94% of targets where LDDT‐PLI > 0.7.

#### 3.1.3 Potential Correlates of Pose Accuracy

**FIGURE 6:**
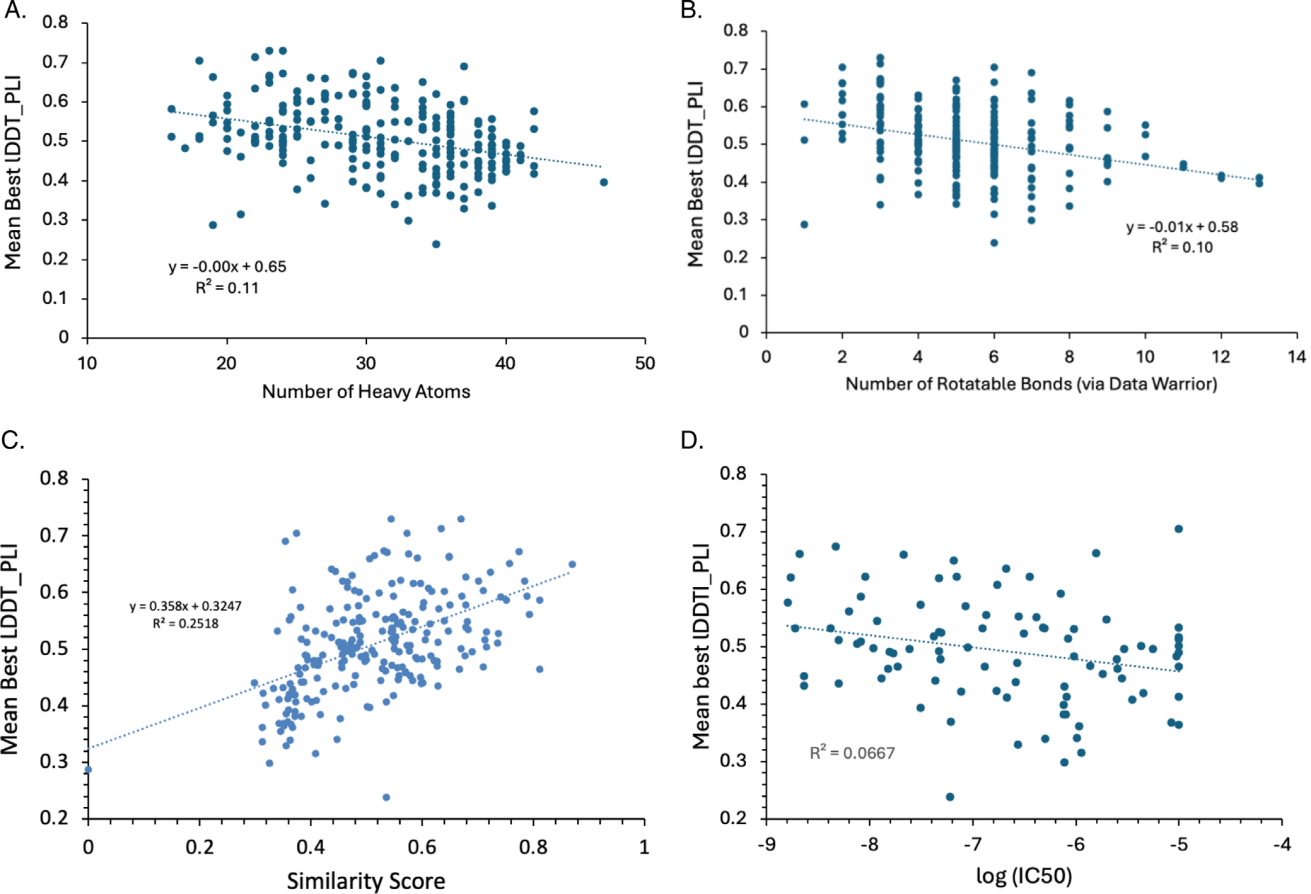
Scatter plots of ligand pose accuracy (measured as mean best lDDT‐PLI across models without skip penalty) averaged across groups for each target, against: (A) number of non‐hydrogen ligand atoms; (B) number of ligand rotatable bonds; (C) SuCOS‐based structure similarity score (Section 2.5) to available protein–ligand structures in the PDB; and (D) ligand affinity given as log (IC50). (A–C) are for all targets, whereas (D) is only for autotaxin targets (L3000). Each point is the average for one ligand across groups. The point with zero similarity corresponds to L5001 (WDR55).

Perhaps also surprising is that there is minimal correlation (*R* ^2^ 0.1) between ligand accuracy and metrics of ligand size (number of non‐hydrogen atoms) and flexibility (number of rotatable bonds), as shown in Figure 6A,B. We also conjectured that ligands with higher similarity to those available to participants in the PDB would be easier to predict, and there is a correlation between the SuCOS similarity score (Section 2.5) and the mean best pose accuracy, but the correlation is not strong, as indicated by a coefficient of determination of 0.25 (Figure 6C). We also considered whether it would be easier to predict the bound poses of higher‐affinity ligands, but this idea is not borne out by the present data (*R* ^2^ 0.07, Figure 6D). Finally, we inspected the ligands that received the highest and lowest mean accuracy across submissions (Figure S7) but did not discern any obvious distinguishing characteristics of the two sets.

#### 3.1.4 Comparison With Baseline Pose Predictors

We used three all‐atom, co‐folding, deep learning methods—AF3, Boltz‐1, and RosettaFold All‐Atom (RFAA)—as well as the physics‐based AD Vina docking method [26] to generate a single pose prediction for each of the CASP16 protein–ligand targets except L5001. These calculations were not conducted in a blinded setting, so the outcomes cannot be viewed as true CASP results. Nonetheless, we ascertained that the deep learning methods had not been trained on data unavailable to the CASP participants. In addition, the baseline methods were applied in an end‐to‐end, automated manner with minimal human input, so it is reasonable to compare them with the CASP group results. Note that, during the challenge period, the publicly available version of AF3 was not able to run this challenge because it could not co‐fold a protein with an arbitrary ligand, only one of a small set of ligands. None of the methods provided results for the four covalent Mpro ligands, and some methods also failed to yield a result for a few additional targets. The total number of omitted or failed predictions is 5, 9, 7, and 5 for AF3, Boltz‐1, RFAA, and AD Vina, respectively.

Treating missing predictions as failures by setting LDDT‐PLI = 0, as done for the CASP groups, we obtain mean LDDT‐PLI scores of 0.80, 0.52, 0.37, and 0.38 for AF3, Boltz‐1, RFAA, and AD Vina, respectively (Table 2). Thus, AF3 outperforms both the Model 1 predictions of all CASP groups (Figure 3A) as well as their best model predictions. Nonetheless, the ClusPro method of CASP group 494 also performs solidly overall and across targets. This method is followed by Boltz‐1, which does well but not exceptionally (compare with Figure 3A), and the RFAA and AD Vina predictions are about average.

**TABLE 2:** Accuracy, in terms of skip‐penalized mean LDDT‐PLI, of each baseline method and CASP group ClusPro (494) across all supertargets and for each individual supertarget.

Method	All	Chymase	Cathepsin G	Autotaxin	Mpro
AF3	0.80	0.88	0.87	0.81	0.63
Boltz‐1	0.53	0.87	0.84	0.48	0.60
RFAA	0.37	0.37	0.19	0.38	0.31
AD Vina	0.38	0.57	0.85	0.36	0.38
ClusPro (494)	0.69	0.72	0.76	0.68	0.72

*Note*: Autotaxin, with 189 targets, dominates the overall averages.

It is not yet clear why Boltz‐1 yields results similar to AF3 for chymase, cathepsin G, and Mpro but underperforms for autotaxin, but we were able to confirm this result with the developers of Boltz‐1 (data not shown), and it has previously been observed that Boltz‐1 is, overall, somewhat less accurate than AF3 in this application [43]. Interestingly, AD Vina performs very competitively for cathepsin G in particular (mean LDDT‐PLI 0.85), but not so well for the other supertargets. Given the sensitivity of docking in general to methodological details, we expect that AD Vina would perform better across the board if our procedure had been tuned (e.g., box size and location, exhaustiveness, treatment of protonation states) based on several targets for each supertarget.

**FIGURE 7:**
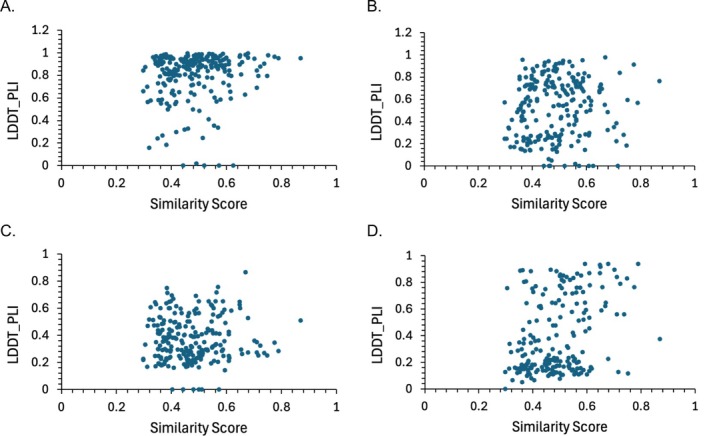
Scatter plots of ligand pose accuracy (measured as LDDT‐PLI) of baseline methods vs. maximum structural similarity with available PDB structures, where similarity is given by the SuCos metric detailed in Methods. Each point is one prediction target (i.e., pose). (A) AlphaFold 3. (B) Boltz‐1. (C) RosettaFold All‐Atom. (D) AutoDock Vina.

Because the deep‐learning methods are trained on structural data in the PDB, we speculated that the accuracy of their predictions would correlate with the similarity of the individual target pose to that of the most similar co‐crystal structure in the PDB. However, this is not the case, as shown in Figure 7, which also demonstrates the same lack of correlation for AD Vina. We probed AF3 further by applying it to the solitary WDR55 target (L5001), for which there was no previously defined binding site and no structure in the PDB with a similarity score (Section 2.5) above 0. Remarkably, AF3 not only placed the ligand in the correct general location but also accurately replicated the crystallographic pose, with LDDT‐PLI of 0.93 and RMSD of 0.48 Å.

### 3.2 Pose Predictions for Incidental Ligands in Pharma Targets

We receive 18 Model 1 submissions with pose predictions for the incidental ligands present in some autotaxin and Mpro structures. These are not drug‐like compounds but small ions and other cosolutes that happen to stick to the surface of the crystallized proteins. We do not think they bind with high affinity; instead, they are found because they are present at high concentrations in the crystals. In Figure S8, we summarize accuracy across these targets in terms of the skip‐penalized mean LDDT‐PLI across each group's Model 1 submissions. The maximum value of this statistic is only 0.39, which is much lower than the maximum of 0.69 achieved for the drug‐like ligands (Figure 3A). Interestingly, though, some of the same methods appear among the best performers, notably Huang‐HUST, kozakovvajda, and Cluspro, although CoDock, which also performed well for the drug‐like ligands, is not among the top for these incidental ligands. We do not find it surprising that accuracy is reduced for the incidental ligands, because their simplicity and lack of chemical features may make it hard to identify the correct pose.

### 3.3 Affinity Predictions

#### 3.3.1 Assessment

The affinity targets provided to CASP16 participants comprised 123 autotaxin cases and 17 chymase cases. However, during the assessment phase of this experiment, we rechecked the publication status of all of the protein–ligand data and discovered that chymase affinity data for chymase affinity targets L1003 and L1010 had been previously disclosed in Roche patents [52, 53, 54, 55] and that another group had published the chymase affinity associated with target L1013 [56]. In addition, the autotaxin affinity data for 15 CASP16 targets, L3009, L3047, L3196, L3197, L3211, L3213, L3214, L3216, L3217, L3219, L3222, L3224, L3225, L3226, and L3229, had been disclosed in the following Roche patents: US10647719, US2023/0312582 A1, US11098048, and US10800786. Therefore, in preparing the assessment below, we omitted these three of the 17 chymase affinity targets and these 15 of the 123 autotaxin affinity targets, so the total number of affinity targets became 122. However, for completeness, Figure S10 in the SI provides an assessment based on all 17 chymase and 123 autotaxin targets, and the results are much the same as those computed without the previously disclosed cases.

We received Stage 1 affinity predictions from 28 groups, where Stage 1 implies predictions made before the experimental poses were made public in order to enable the Stage 2 challenge. We assessed the overall affinity prediction accuracy of groups in terms of the *N*‐weighted Kendall's *τ* across both sets of targets as $\kappa_{N}=\frac{N_{A}\tau_{A}+N_{C}\tau_{C}}{N_{A}+N_{C}}$, where *N*_*x*_ is the number of affinities submitted for autotaxin (*N*_*A*_ = 108) or chymase (*N*_*C*_ = 14), and *τ*_*x*_ is Kendall's *τ* computed for *x* = *A*, *C*.

**FIGURE 8:**
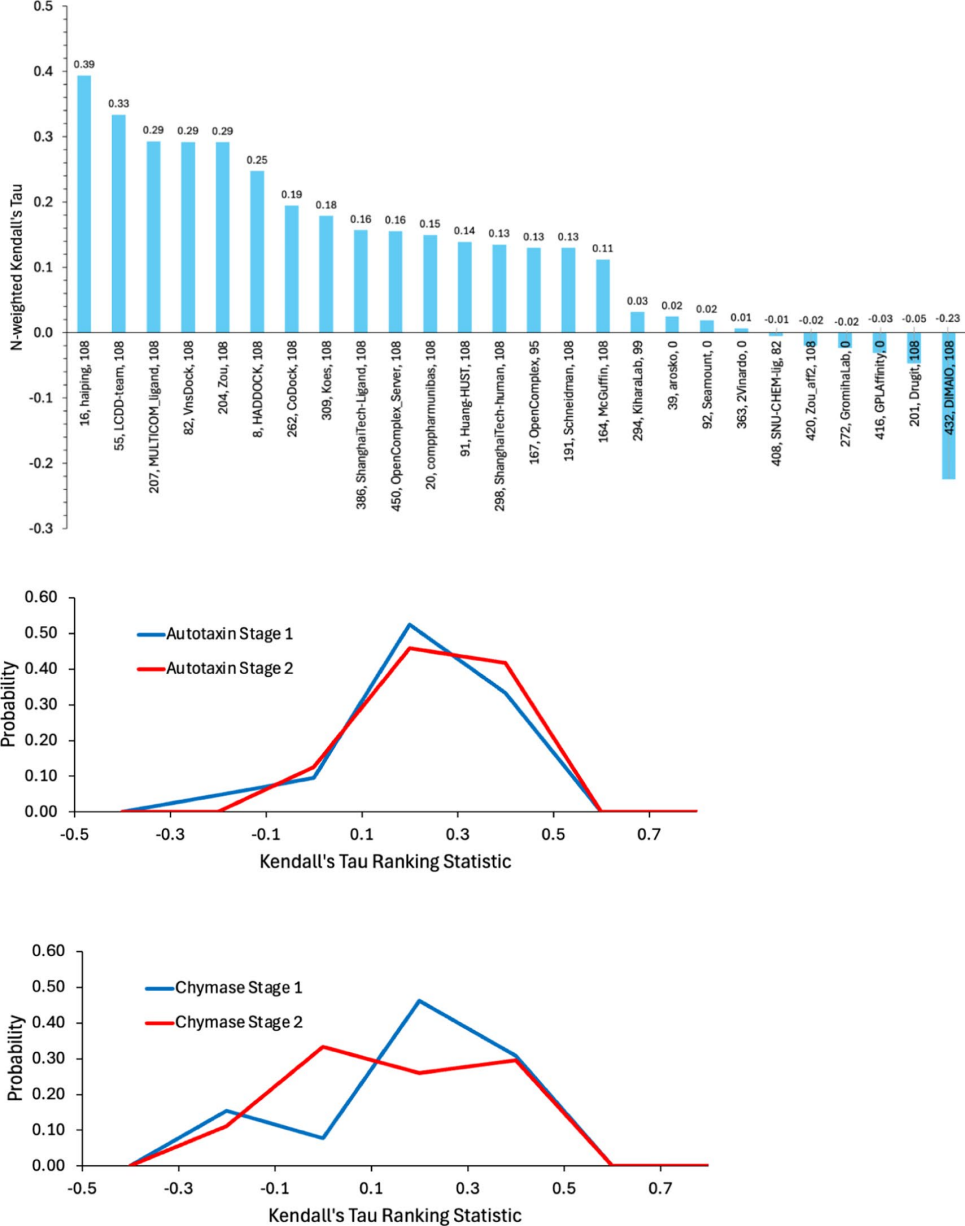
Assessment of affinity predictions. (A) Accuracy assessment of Stage 1 affinity predictions in terms of *N*‐weighted Kendall's *τ* of each group's Model 1 predictions. Labels indicate group ID, method name, and number of affinities ranked by the group. (B, C) Distribution of accuracies of Stage 1 (blue) versus Stage 2 (red) affinity predictions via normalized histograms of Kendall's *τ* ranking statistics for (B) autotaxin and (C) chymase. There were 21 Stage 1 and 24 Stage 2 submissions for autotaxin and 28 Stage 1 and 27 Stage 2 submissions for chymase.

Encouragingly, most of the affinity predictions correlate positively with the experimental data, though performance quality varies widely (Figure 8A). The groups that provide the best affinity predictions are not the same as those that provide the best pose predictions (Section 3.1.2). Haiping (group 16) stands out ($\kappa_{N}$ = 0.39) and is followed by four other groups with $\kappa_{N}$ > 0.25, namely LCDD‐team (55), MULTICOM_ligand (207), VnsDock (82), and Zou (204). MULTICOM_ligand, which also shows up among the better pose predictions, uses multiple DNN‐based methods, including NeuralPlexer [57] and DiffDock [58], in combination. Interestingly, we observe no significant correlation between the accuracy of a group's predictions for autotaxin and those for chymase (Figure S9): The groups that performed best on autotaxin did not do particularly well for chymase, and vice versa, except for Haiping, which did well on both. This method uses graph neural networks trained on the PDBbind [59] database; one approach uses a single graph for the full protein–ligand complex structure, whereas another builds separate graphs for the protein binding pocket and the ligand. Intriguingly, the maximum value of Kendall's *τ* observed here, about 0.4, is similar to the values of Kendall's *τ* observed in the affinity‐prediction components of the D3R Grand Challenges [14].

**FIGURE 9:**
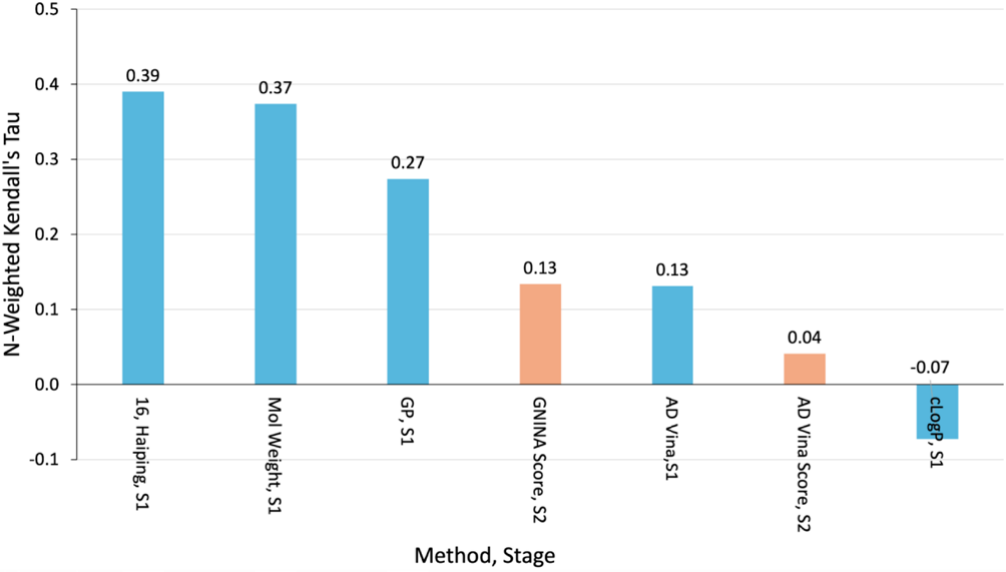
*N*‐weighted Kendall's *τ* for each baseline affinity predictor. Blue columns are for the Stage 1 (S1) targets; orange columns are for Stage 2 (S2).

In order to put these results into context, we computed the expectation values of Kendall's *τ* for a perfect prediction method against experimental data subject to ~3× uncertainty in the measured IC50 (Section 2.3). For chymase, the mean resampled Kendall's *τ* is 0.54 (SD: 0.11), and for autotaxin, the value is 0.76 (SD: 0.02). The difference between these outcomes traces to the fact that the IC50s for chymase vary over a much smaller range (400×) than those for autotaxin (8700×), and a larger range makes the ranking more stable in the face of 3× experimental uncertainty. Weighting these values by the number of measurements in each dataset, as done in preparing the summary data in Figure 9A, we obtain an expected mean best Kendall's *τ* of 0.73. This is well above the maximum achieved value of 0.39 (Figure 9A), so there is still considerable room for improvement in this challenge component. Note that the fact that CASP participants came close to the theoretical maximum value of Kendall's *τ* for chymase does not imply that these predictions were more successful, only that the chymase challenge is less capable of resolving levels of accuracy, due, again, to the small range of experimental data. We can also use this approach to estimate the error in the calculations. Thus, if we assign an error of 30× in IC50 (2 kcal/mol in binding free energy), we obtain a mean expected best Kendall's *τ* of 0.43 for autotaxin, which is similar to the best result in Figure 8A. This suggests that the best affinity prediction method has an effective uncertainty of ~2 kcal/mol in binding free energy.

In Stage 2, participants could use the experimental structures in making their affinity predictions. Structures were available for all 17 of the originally posed chymase targets, so 14 were used following the removal of three previously disclosed data points. Structures were available for 93 of the original autotaxin affinity targets, and 89 undisclosed affinities with associated cocrystal structures were available for Stage 2. It is evident from the histograms of Kendall's *τ* values over all groups (Figure 8B,C) that knowledge of the structures did not lead to a shift toward greater accuracy.

#### 3.3.2 Comparison With Baseline Affinity Predictors

To provide context for the results obtained by CASP participants on the affinity prediction tasks, we ran several baseline predictors. First, as in D3R, we ranked compounds simply by MolW and log of the computed octanol–water partition coefficient (cLogP), with higher values considered conducive to binding. Second, we used a classical regression model (GPR) fitted for each supertarget individually using data from ChEMBL. Third, for the Stage 1 targets, we used AD Vina to dock each compound to its respective target and considered whether the docking scores correlated with affinity, whereas for the Stage 2 targets, we computed AD Vina scores and GNINA [40] scores for the available crystal structures.

The MolW of the ligand correlates with experiment almost as well as the best CASP predictions (Figure 9), while cLogP has an inverse correlation. A similar pattern was observed for the BACE 1 target in D3R's Grand Challenge 4, but in Grand Challenge 2, cLogP performed well and MolW performed poorly for FXR. Thus, although these descriptors sometimes do very well, they are unreliable. The Gaussian Process model put in a credible but not outstanding performance, while the docking/scoring methods did not fare well (Figure 9). Interestingly, scoring crystallographic poses with AD Vina in Stage 2 gave lower accuracy than docking and scoring with AD Vina in Stage 1. It should again be emphasized that these calculations were run in a uniform manner with essentially no human intervention, and better results might be obtained with some tuning on, e.g., a subset of the ligands.

### 3.4 Reliability of Pose Accuracy Self‐Assessments

**FIGURE 10:**
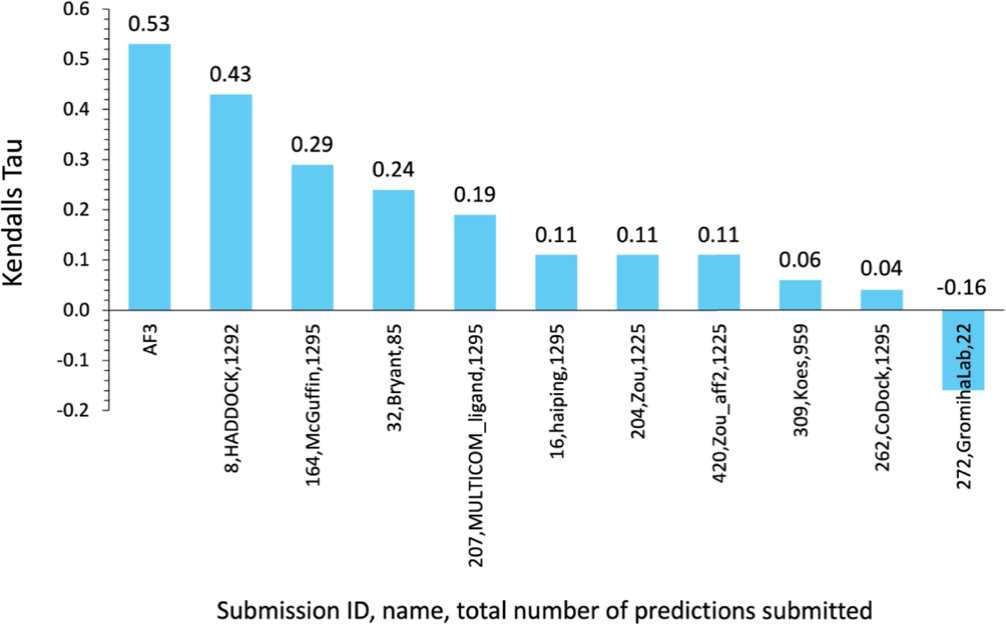
Correlations (Kendall's *τ*) of pose prediction accuracy (LDDT‐PLI) with reliability score (LScore).

We encouraged participants to indicate their confidence in each pose prediction with a score, called the LScore, where the minimum value of 0 indicates low confidence in the prediction and the maximum of 1 indicates high confidence. We assessed the reliability of LScores by looking for a correlation between LScore and prediction accuracy as measured by LDDT‐PLI on all models. Correlation was measured in terms of Kendall's *τ* without any penalty for skipped predictions. As shown in Figure 10, few groups provided LScores, and of these, HADDOCK (8) performed relatively well, while the other correlations were rather modest. Interestingly, AlphaFold 3 performed best, with a Kendall's *τ* of 0.53, when we interpreted pLDDT from AlphaFold 3 as LScore.

## 4 Discussion

This first CASP challenge to include pose and affinity predictions for drug‐like ligands attracted a strong pool of participants (30 labs) deploying a range of different methods (34 CASP groups). We observed a wide range of accuracy, with some methods decidedly more accurate than others. However, a challenge based on different targets would undoubtedly have led to a somewhat different ranking among the higher‐performing methods, if only due to chance. Specific characteristics that might lead to variations in performance across methods include the amount of data already available for the target protein and the character of the binding site, for example, flexible versus rigid.

That said, some themes do emerge. All of the leading pose‐prediction methods are at least partly template‐based, which means that they seek to model the protein–ligand structure on existing similar co‐crystal structures available in the PDB. This result is consistent with the early conclusion of the Drug Design Data Resource (D3R) project that “there appears to be considerable potential for creation of automated software and workflows that go beyond pure docking and scoring by automatically collecting and effectively using available information, such as crystal structures … to generate enhanced pose predictions…” [60]. Interestingly, the affinity prediction method that yielded the best performance here scores ligands with a graph neural network trained against available experimental data, rather than using a physics‐based scoring function. Thus, the leading pose‐prediction and affinity‐prediction methods make extensive use of the existing public dataset of structures and affinities. Such approaches may be particularly applicable to the protein–ligand challenges posed in CASP16, because the protein targets are already well characterized, with many data available in the public domain. We also assessed several easily automated baseline pose‐prediction and affinity‐prediction methods. Perhaps the most striking result is that AlphaFold 3 did very well at pose predictions. This outcome may, again, reflect the fact that the main protein targets here are already well represented in the PDB. In future challenges, we hope that some less well‐characterized systems may be included among the targets in order to better probe the generalizability of pose‐prediction methods to novel drug discovery targets.

Another broad result is that the accuracy of the affinity predictions did not improve when, in Stage 2, we made the experimental co‐crystal structures available to the participants. This result is consistent with most outcomes in the D3R challenges and suggests that the accuracy of the affinity predictions used here is still limited primarily by the scoring models used. We would not be surprised if more refined affinity prediction methods, such as simulation‐based free energy calculations [44, 45], proved more sensitive to the accuracy of the starting pose.

Participants were not particularly good at assessing the reliability of their pose predictions in advance, as evidenced by the generally low correlations between pose accuracy and LScore. This result is arguably consistent with the lack of correlation we found between pose accuracy and metrics of ligand complexity, such as ligand size. Users of pose‐ and affinity‐prediction methods would presumably value some indication of the reliability of the predictions, so this would appear to be an important—if difficult—direction for future research.

It is interesting to compare the CASP16 experience with those of the D3R Grand Challenges 2, 3, and 4, which were held 2016–2019 and attracted predictions from 49, 28, and 51 labs, respectively. However, it is not clear that the CASP16 pose‐prediction results reflect significant methodological improvement since D3R, especially given that the protein–ligand targets were different and that there was considerable target‐to‐target variation even within a single challenge. For affinity predictions, it seems clear that the best Stage 1 CASP16 results are no better than the typical best Stage 1 D3R result, which averaged Kendall's *τ* of 0.48, with min/max of 0.21 and 0.71.

Given that pose‐prediction challenges can use well‐resolved crystal structures of protein–ligand complexes, there seems to be little ambiguity in defining this field's ultimate goal of achieving near 100% success in predicting poses to within the commonly used cutoff of 2.0–2.5 Å—values chosen because this quality of prediction is needed in CADD applications. Similarly, we would look for affinity methods to approach the theoretical maximum value of Kendall's *τ* for the given data set, noting that this value depends on the range and distribution of the data points and the level of experimental error. Currently, there is still considerable room for improvement on both fronts.

As a number of computational methods currently used in industrial drug discovery did not appear in CASP 16, it would be helpful to the field if a wider set of methods could be applied by experienced users in future rounds. For example, although simulation‐based free energy methods are time‐consuming, they can be quite accurate and, indeed, did particularly well in the last D3R challenge [14]. It may be worth crafting a specialized subchallenge that is tailored for such methods, as previously done [14]. In addition, many different ways of using DNNs for protein–drug modeling are being explored worldwide, and it will be exciting to see how these perform after another 2 years of development.

## 5 Conclusions

The CASP16 protein–ligand challenge summarized here represents the first assessment of pose and affinity predictions for drug‐like compounds within the CASP framework. Predictions submitted by 30 research laboratories varied considerably in their accuracy, with the most accurate pose predictions coming from template‐based approaches based explicitly on existing structures available in the PDB. Similarly, the most successful affinity prediction method employed graph neural networks trained on experimental data rather than physics‐based scoring functions. Access to experimental structures in Stage 2 did not improve affinity predictions, consistent with observations in the prior D3R challenges and suggesting that current scoring models are a key limiting factor in affinity prediction accuracy. We also tested several baseline methods and found that AlphaFold 3 did particularly well, outperforming all participant methods. These results highlight both the progress made in computational protein–ligand modeling and the substantial room for improvement in this space. Future CASP protein–ligand challenges would benefit from the inclusion of less well‐characterized target systems to better evaluate method generalizability, and from the application of other computational approaches currently used in industrial drug discovery settings, such as simulation‐based free energy methods.

## Author Contributions

**Michael K. Gilson:** conceptualization, investigation, writing – original draft, methodology, validation, visualization, writing – review and editing, formal analysis, data curation, supervision. **Jerome Eberhardt:** conceptualization, methodology, validation, visualization, investigation, writing – review and editing, data curation, software, formal analysis. **Peter Škrinjar:** investigation, methodology. **Janani Durairaj:** investigation, methodology. **Xavier Robin:** conceptualization, investigation, writing – review and editing, validation, visualization, software, formal analysis, data curation, methodology. **Andriy Kryshtafovych:** conceptualization, investigation, writing – review and editing, validation, visualization, project administration, resources, supervision, data curation.

## Conflicts of Interest

M.K.G. has an equity interest in and is a cofounder and scientific advisor of VeraChem LLC. He is also on the scientific advisory boards of Denovicon Therapeutics, In Cerebro, Cold Start Therapeutics, and Beren Therapeutics.

## Supporting information


**Table S1:** FASTA sequences of the target proteins in the CASP16 ligand–protein prediction challenge.
**Table S2:** Skip‐penalized mean LDDT‐PLI results for Model 1 predictions of all groups and supertargets. All is the combined result shown in Figure 5A.
**Figure S1:** Scatter plot of ligand pose accuracy (as RMSD) against binding site structure accuracy (as BB‐RMSD).
**Figure S2:** (A) Histogram of number of groups (*y*‐axis) predicting a given number of targets (*x*‐axis). Bin width is 20; the first bin is 0–20, and the last bin is 220–240. (B) Scatter plot of mean accuracy over all targets for each group versus the number of targets.
**Figure S3:** Two examples of predicted ligand poses (magenta) and corresponding crystallographic poses (green) for the chymase supertarget (left: L1008 and right: L1009) with RMSD 2.5 Å and LDDT_PLI of 0.83 and 0.68, respectively.
**Figure S4:** Scatter plot of skip‐penalized success rate (fraction of predictions with RMSD of at most 2.5 Å) versus skip‐penalized LDDT‐PLI for each group.
**Figure S5:** Scatter plots of mean accuracy (LDDT‐PLI) of groups for pairs of supertargets, computed without penalizing skipped predictions. Each dot represents one group. A group was included here only if it contained predictions for at least 17 chymase predictions, 167 autotaxin predictions, and 15 Mpro predictions. Cathepsin G and WDR55, with only 2 and 1 targets apiece, are omitted here.
**Figure S6:** Scatter plot of the mean best LDDT‐PLI across models versus the mean Model 1 LDDT‐PLI. Each dot is one group.
**Figure S7:** Four ligands with the highest (0.70–0.73) and lowest (0.24–0.31) mean best LDDT‐PLI across all submitted models, without skip penalty, averaged across groups. The target IDs of the high‐accuracy ligands are 1010, 4028, 4027, and 3192. The target IDs of the low‐accuracy ligands are 3083, 3058, 5001, and 3109.
**Figure S8:** Pose prediction accuracy, by group, for the incidental (non‐drug‐like) ligands.
**Figure S9:** Scatter plot of Kendall's τ for chymase (vertical axis) versus autotaxin (horizontal), computed without any skip penalty. Each point represents one group. The pale blue point at the top right corresponds to group 16.
**Figure S10:** Assessment of affinity predictions in the manner of Figure 8, but including the affinity targets found, during analysis, to have been previously published (see Section 3.3.1).

## Data Availability

The data that support the findings of this study are openly available in CASP Prediction Center at https://predictioncenter.org/casp16/results.cgi?tr_type=ligand.
