# Supplementary material for: Assessment of Pharmaceutical Protein–Ligand Pose and Affinity Predictions in CASP16
Source: Proteins. 2025 Oct 4;94(1):249–66. doi: 10.1002/prot.70061 (PMC12750038; doi:10.1002/prot.70061)
Supplement: Supplementary file 1 — Table S1: FASTA sequences of the target proteins in the CASP16 ligand–protein prediction challenge. Table S2: Skip‐penalized mean LDDT‐PLI results for Model 1 predictions of all groups and supertargets. All is the combined result shown in Figure 5A. Figure S1: Scatter plot of ligand pose accuracy (as RMSD) against binding site structure accuracy (as BB‐RMSD). Figure S2: (A) Histogram of number of groups (y‐axis) predicting a given number of targets (x‐axis). Bin width is 20; the first bin is 0–20, and the last bin is 220–240. (B) Scatter plot of mean accuracy over all targets for each group versus the number of targets. Figure S3: Two examples of predicted ligand poses (magenta) and corresponding crystallographic poses (green) for the chymase supertarget (left: L1008 and right: L1009) with RMSD 2.5 Å and LDDT_PLI of 0.83 and 0.68, respectively. Figure S4: Scatter plot of skip‐penalized success rate (fraction of predictions with RMSD of at most 2.5 Å) versus skip‐penalized LDDT‐PLI for each group. Figure S5: Scatter plots of mean accuracy (LDDT‐PLI) of groups for pairs of supertargets, computed without penalizing skipped predictions. Each dot represents one group. A group was included here only if it contained predictions for at least 17 chymase predictions, 167 autotaxin predictions, and 15 Mpro predictions. Cathepsin G and WDR55, with only 2 and 1 targets apiece, are omitted here. Figure S6: Scatter plot of the mean best LDDT‐PLI across models versus the mean Model 1 LDDT‐PLI. Each dot is one group. Figure S7: Four ligands with the highest (0.70–0.73) and lowest (0.24–0.31) mean best LDDT‐PLI across all submitted models, without skip penalty, averaged across groups. The target IDs of the high‐accuracy ligands are 1010, 4028, 4027, and 3192. The target IDs of the low‐accuracy ligands are 3083, 3058, 5001, and 3109. Figure S8: Pose prediction accuracy, by group, for the incidental (non‐drug‐like) ligands. Figure S9: Scatter plot of Kendall's τ for chymase (vertical a [file PROT-94-249-s001.docx]

Assessment of Pharmaceutical Protein-Ligand Pose and Affinity Predictions in CASP16

# **Supporting Information**

Michael K. Gilson^1*^, Jerome Eberhardt^2,3^, Peter Škrinjar^2,3^, Janani Durairaj^2,3^, Xavier Robin^2,3^, and Andriy Kryshtafovych^4^

1. Skaggs School of Pharmacy and Pharmaceutical Sciences, University of California San Diego, La Jolla, CA, USA.
2. SIB Swiss Institute of Bioinformatics, Basel 4056, Switzerland
3. Biozentrum, University of Basel, Basel 4056, Switzerland
4. Genome Center, University of California, Davis, CA, USA

* To whom correspondence should be addressed: mgilson@ucsd.edu

#

#

| \| **Protein** \| **FASTA sequence** \| \| --- \| --- \| \| Chymase \| >L1000 series, Chymase P23946 isoform 1 - 2 mutations: ECK -> ESK, QFN -> QKN  MLLLPLPLLLFLLCSRAEAGEIIGGTESKPHSRPYMAYLEIVTSNGPSKFCGGFLIRRNFVLTAAHCAGRSITVTLGAHNITEEEDTWQKLEVIKQFRHPKYNTSTLHHDIMLLKLKEKASLTLAVGTLPFPSQKNFVPPGRMCRVAGWGRTGVLKPGSDTLQEVKLRLMDPQACSHFRDFDHNLQLCVGNPRKTKSAFKGDSGGPLLCAGVAQGIVSYGRSDAKPPAVFTRISHYRPWINQILQAN \| \| Cathepsin G \| >L2000 series, Cathepsin G  MQPLLLLLAFLLPTGAEAGEIIGGRESRPHSRPYMAYLQIQSPAGQSRCGGFLVREDFVLTAAHCWGSNINVTLGAHNIQRRENTQQHITARRAIRHPQYNQRTIQNDIMLLQLSRRVRRNRNVNPVALPRAQEGLRPGTLCTVAGWGRVSMRRGTDTLREVQLRVQRDRQCLRIFGSYDPRRQICVGDRRERKAAFKGDSGGPLLCNNVAHGIVSYGKSSGVPPEVFTRVSSFLPWIRTTMRSFKLLDQMETPL \| \| Autotaxin \| >L3000 series, Autotaxin  FTASRIKRAEWDEGPPTVLSDSPWTATSGSCKGRCFELQEVGPPDCRCDNLCKSYSSCCHDFDELCLKTARGWECTKDRCGEVRNEENACHCSEDCLSRGDCCTNYQVVCKGESHWVDDDCEEIKVPECPAGFVRPPLIIFSVDGFRASYMKKGSKVMPNIEKLRSCGTHAPYMRPVYPTKTFPNLYTLATGLYPESHGIVGNSMYDPVFDASFHLRGREKFNHRWWGGQPLWITATKQGVRAGTFFWSVSIPHERRILTILQWLSLPDNERPSVYAFYSEQPDFSGHKYGPFGPEMTNPLREIDKTVGQLMDGLKQLRLHRCVNVIFVGDHGMEDVTCDRTEFLSNYLTNVDDITLVPGTLGRIRAKSINNSKYDPKTIIAALTCKKPDQHFKPYMKQHLPKRLHYANNRRIEDIHLLVDRRWHVARKPLDVYKKPSGKCFFQGDHGFDNKVNSMQTVFVGYGPTFKYRTKVPPFENIELYNVMCDLLGLKPAPNNGTHGSLNHLLRTNTFRPTMPDEVSRPNYPGIMYLQSEFDLGCTCDDKVEPKNKLEELNKRLHTKGSTKERHLLYGRPAVLYRTSYDILYHTDFESGYSEIFLMPLWTSYTISKQAEVSSIPEHLTNCVRPDVRVSPGFSQNCLAYKNDKQMSYGFLFPPYLSSSPEAKYDAFLVTNMVPMYPAFKRVWAYFQRVLVKKYASERNGVNVISGPIFDYNYDGLRDTEDEIKQYVEGSSIPVPTHYYSIITSCLDFTQPADKCDGPLSVSSFILPHRPDNDESCNSSEDESKWVEELMKMHTARVRDIEHLTGLDFYRKTSRSYSEILTLKTYLHTYESEIGGRHHHHHHHH \| \| Mpro \| >L4000 series, MPro  SGFRKMAFPSGKVEGCMVQVTCGTTTLNGLWLDDVVYCPRHVICTSEDMLNPNYEDLLIRKSNHNFLVQAGNVQLRVIGHSMQNCVLKLKVDTANPKTPKYKFVRIQPGQTFSVLACYNGSPSGVYQCAMRPNFTIKGSFLNGSCGSVGFNIDYDCVSFCYMHHMELPTGVHAGTDLEGNFYGPFVDRQTAQAAGTDTTITVNVLAWLYAAVINGDRWFLNRFTTTLNDFNLVAMKYNYEPLTQDHVDILGPLSAQTGIAVLDMCASLKELLQNGMNGRTILGSALLEDEFTPFDVVRQCSGVTFQ \| \| WDR55 \| >L5001, WDR55  MDRTCEERPAEDGSDEEDPDSMEAPTRIRDTPEDIVLEAPASGLAFHPARDLLAAGDVDGDVFVFSYSCQEGETKELWSSGHHLKACRAVAFSEDGQKLITVSKDKAIHVLDVEQGQLERRVSKAHGAPINSLLLVDENVLATGDDTGGICLWDQRKEGPLMDMRQHEEYIADMALDPAKKLLLTASGDGCLGIFNIKRRRFELLSEPQSGDLTSVTLMKWGKKVACGSSEGTIYLFNWNGFGATSDRFALRAESIDCMVPVTESLLCTGSTDGVIRAVNILPNRVVGSVGQHTGEPVEELALSHCGRFLASSGHDQRLKFWDMAQLRAVVVDDYRRRKKKGGPLRALSSKTWSTDDFFAGLREEGEDSMAQEEKEETGDDSD \| |
| --- | --- | --- | --- | --- | --- | --- | --- | --- | --- | --- | --- | --- |
| Table S1. FASTA sequences of the target proteins in the CASP16 ligand-protein prediction challenge. |

|  | **Skip-Penalized Mean LDDT-PLI** | | | | | |  |
| --- | --- | --- | --- | --- | --- | --- | --- |
|  | **Chymase** | **Cathepsin G** | **Autotaxin** | **Mpro** | **WDR55** |  |  |
| **Group ID, Method Name, Number of predictions** | **L1000** | **L2000** | **L3000** | **L4000** | **L5000** | **All** |  |
| 494,ClusPro,229 | 0.76 | 0.72 | 0.68 | 0.72 | 0.00 | 0.69 |  |
| 274,kozakovvajda,229 | 0.75 | 0.72 | 0.68 | 0.72 | 0.00 | 0.69 |  |
| 262,CoDock,229 | 0.65 | 0.86 | 0.59 | 0.70 | 0.55 | 0.60 |  |
| 91,Huang-HUST,228 | 0.73 | 0.41 | 0.59 | 0.55 | 0.00 | 0.59 |  |
| 207,MULTICOM_ligand,229 | 0.83 | 0.75 | 0.59 | 0.41 | 0.00 | 0.59 |  |
| 432,DIMAIO,229 | 0.77 | 0.85 | 0.54 | 0.55 | 0.73 | 0.56 |  |
| 420,Zou_aff2,224 | 0.66 | 0.54 | 0.53 | 0.53 | 0.00 | 0.54 |  |
| 55,LCDD-team,229 | 0.62 | 0.55 | 0.51 | 0.61 | 0.00 | 0.52 |  |
| 204,Zou,224 | 0.62 | 0.46 | 0.50 | 0.51 | 0.00 | 0.51 |  |
| 408,SNU-CHEM-lig,211 | 0.75 | 0.86 | 0.42 | 0.66 | 0.00 | 0.46 |  |
| 8,HADDOCK,228 | 0.36 | 0.67 | 0.44 | 0.36 | 0.00 | 0.43 |  |
| 294,KiharaLab,213 | 0.66 | 0.83 | 0.34 | 0.47 | 0.03 | 0.38 |  |
| 309,Koes,228 | 0.68 | 0.88 | 0.32 | 0.49 | 0.00 | 0.37 |  |
| 16,haiping,228 | 0.35 | 0.49 | 0.38 | 0.25 | 0.00 | 0.36 |  |
| 82,VnsDock,226 | 0.38 | 0.00 | 0.38 | 0.14 | 0.00 | 0.35 |  |
| 298,ShanghaiTech-human,227 | 0.22 | 0.56 | 0.32 | 0.48 | 0.00 | 0.33 |  |
| 227,KUMC,229 | 0.45 | 0.74 | 0.30 | 0.36 | 0.00 | 0.32 |  |
| 191,Schneidman,228 | 0.65 | 0.61 | 0.26 | 0.31 | 0.00 | 0.29 |  |
| 386,ShanghaiTech-Ligand,207 | 0.22 | 0.56 | 0.32 | 0.00 | 0.00 | 0.28 |  |
| 167,OpenComplex,206 | 0.31 | 0.54 | 0.23 | 0.43 | 0.00 | 0.26 |  |
| 201,Drugit,228 | 0.45 | 0.18 | 0.23 | 0.36 | 0.00 | 0.25 |  |
| 164,McGuffin,228 | 0.27 | 0.04 | 0.20 | 0.33 | 0.00 | 0.21 |  |
| 450,OpenComplex_Server,228 | 0.19 | 0.54 | 0.17 | 0.43 | 0.00 | 0.20 |  |
| 39,arosko,40 | 0.59 | 0.15 | 0.00 | 0.55 | 0.70 | 0.10 |  |
| 20,comppharmunibas,59 | 0.13 | 0.66 | 0.05 | 0.35 | 0.00 | 0.09 |  |
| 464,PocketTracer,229 | 0.14 | 0.73 | 0.07 | 0.00 | 0.00 | 0.07 |  |
| 363,2Vinardo,17 | 0.81 | 0.00 | 0.00 | 0.00 | 0.00 | 0.06 |  |
| 474,CCB-AlGDock,20 | 0.00 | 0.00 | 0.00 | 0.31 | 0.00 | 0.03 |  |
| 272,GromihaLab,22 | 0.11 | 0.15 | 0.02 | 0.00 | 0.00 | 0.02 |  |
| 32,Bryant,19 | 0.30 | 0.20 | 0.00 | 0.00 | 0.00 | 0.02 |  |
| 416,GPLAffinity,17 | 0.31 | 0.00 | 0.00 | 0.00 | 0.00 | 0.02 |  |
| 92,Seamount,8 | 0.25 | 0.38 | 0.00 | 0.00 | 0.00 | 0.02 |  |
| 189,LCBio,2 | 0.00 | 0.77 | 0.00 | 0.00 | 0.00 | 0.01 |  |
| 261,UNRES,2 | 0.00 | 0.40 | 0.00 | 0.00 | 0.00 | 0.00 |  |
| **Number of Targets** | **17** | **2** | **189** | **20** | **1** | **229** |  |

Table S2. Skip-penalized mean LDDT-PLI results for Model 1 predictions of all groups and supertargets. **All** is the combined result shown in Figure 5A.

| 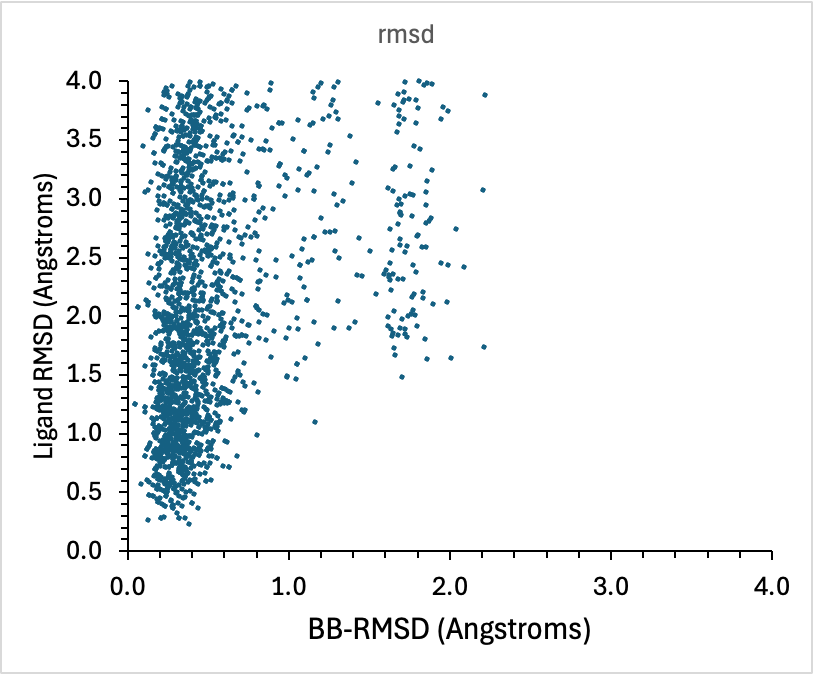 |
| --- |
| Figure S1. Scatter plot of ligand pose accuracy (as RMSD) against binding site structure accuracy (as BB-RMSD). |

| A.  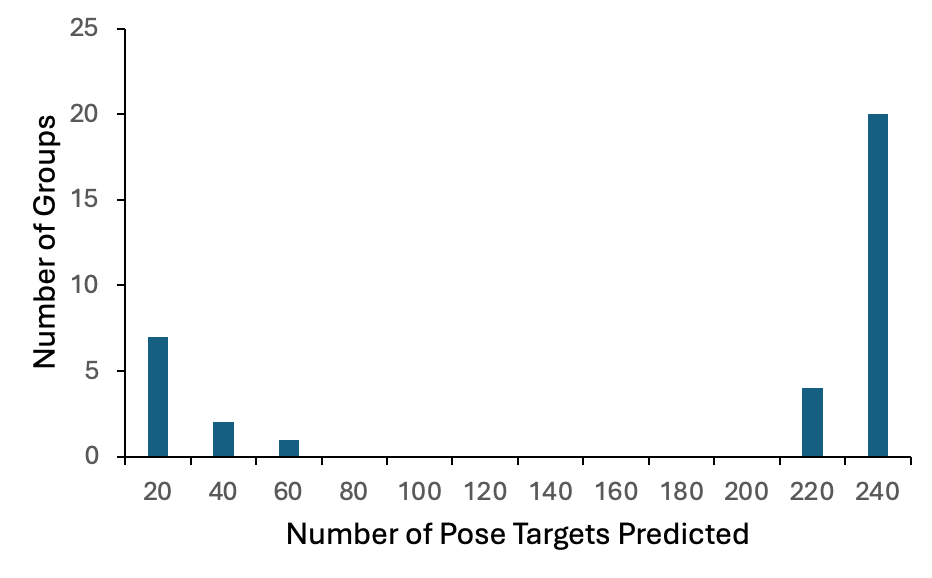 |
| --- |
| B.  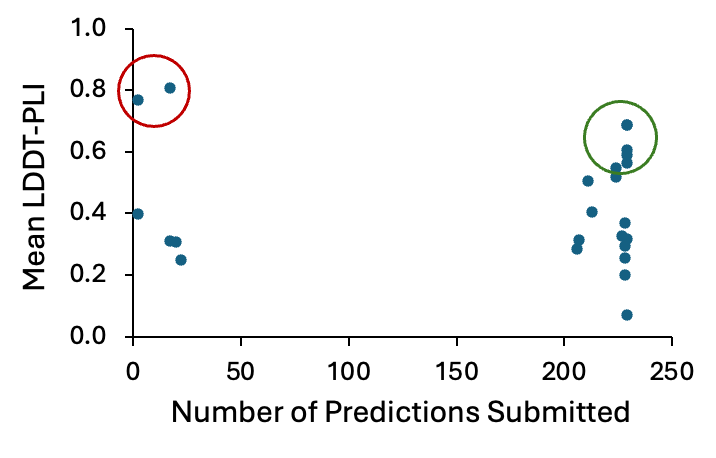 |
| Figure S2. A. Histogram of number of groups (y-axis) predicting a given number of targets (x-axis). Bin width is 20; the first bin is 0-20, and the last bin is 220-240. B. Scatter plot of mean accuracy over all targets for each group versus the number of targets. |

| 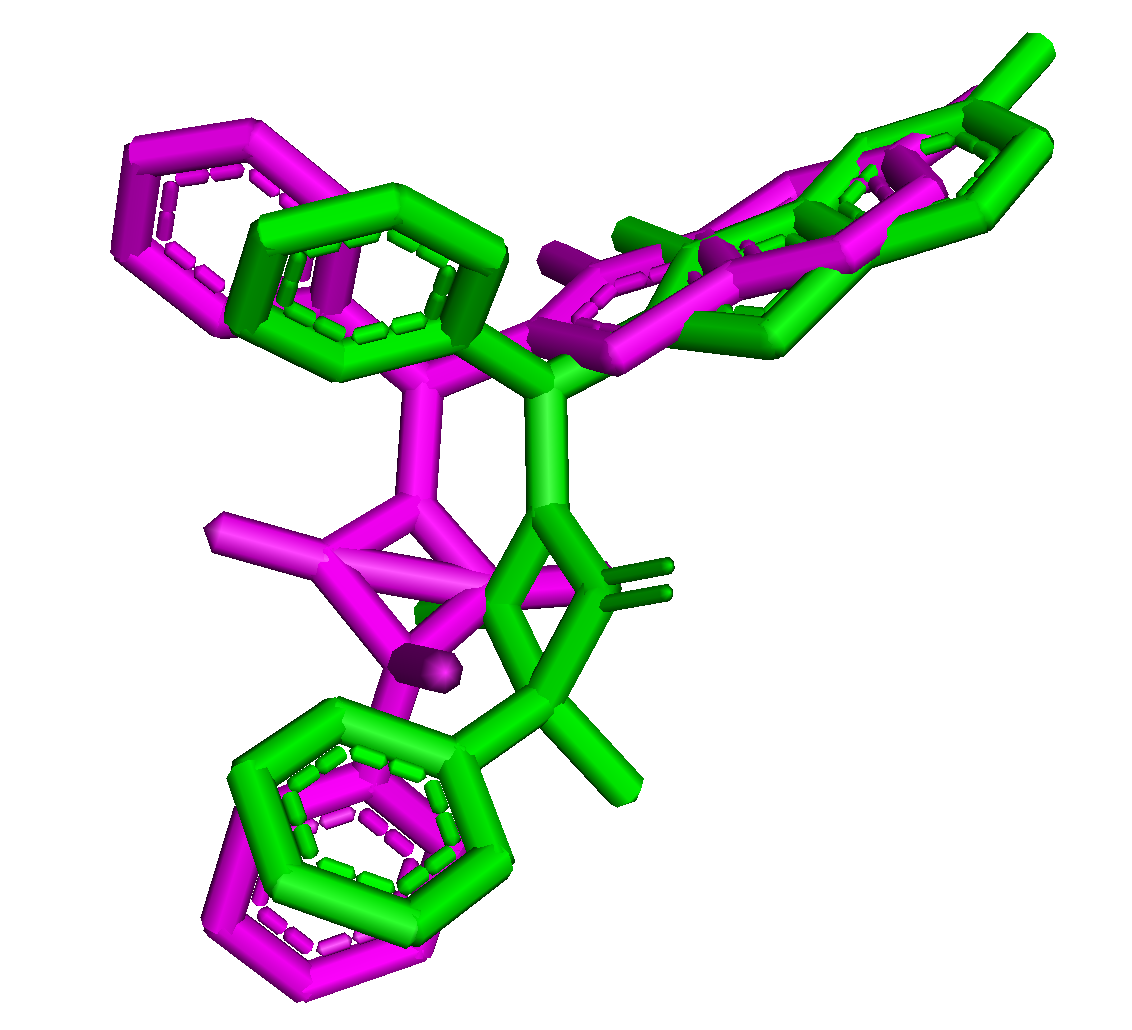 | 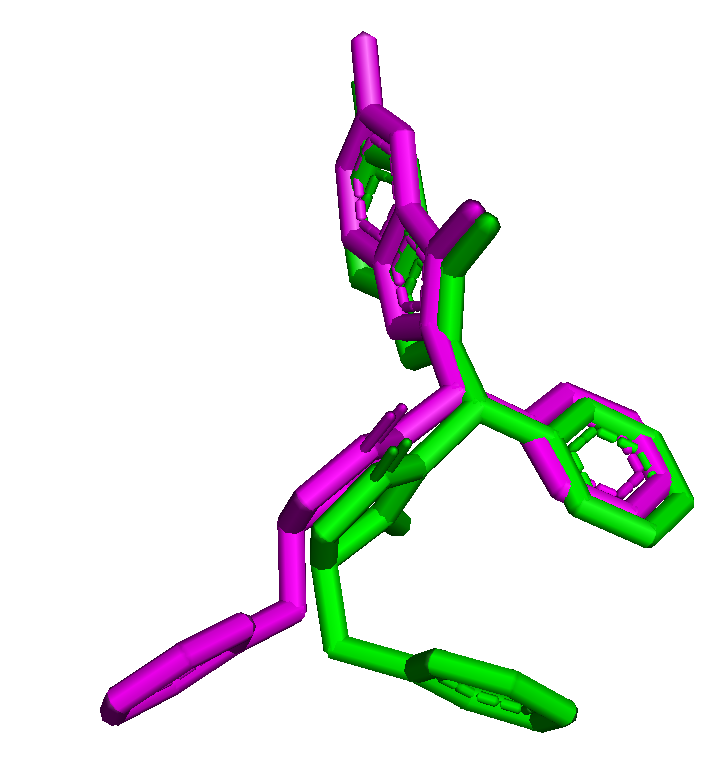 |
| --- | --- |
| Figure S3. Two examples of predicted ligand poses (magenta) and corresponding crystallographic poses (green) for the chymase supertarget (left L1008 and right L1009) with RMSD 2.5 Angstroms and LDDT_PLI of 0.83 and 0.68, respectively. | |

| 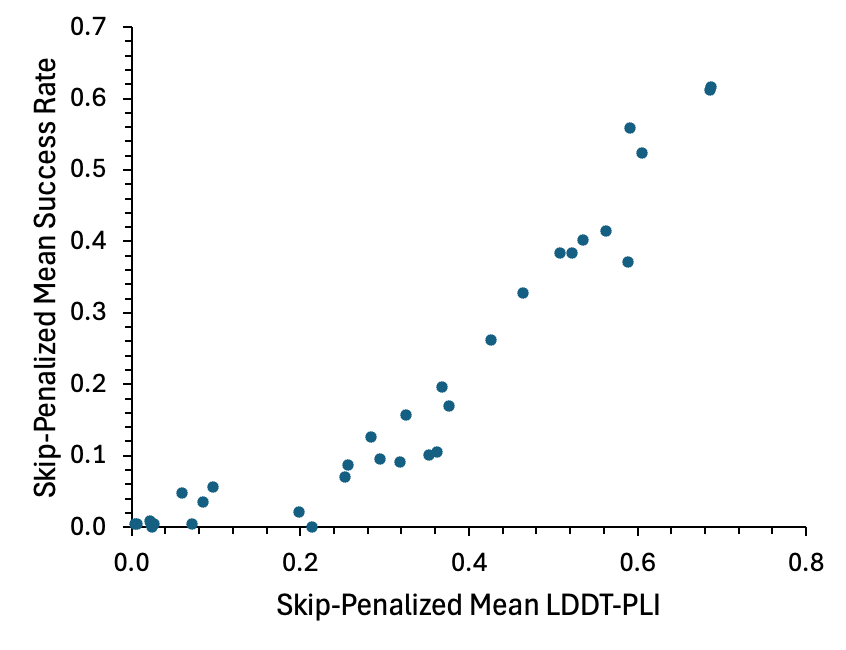 |
| --- |
| Figure S4. Scatter plot of skip-penalized success rate (fraction of predictions with RMSD of at most 2.5 Angstroms) vs. skip-penalized LDDT-PLI for each group. |

| 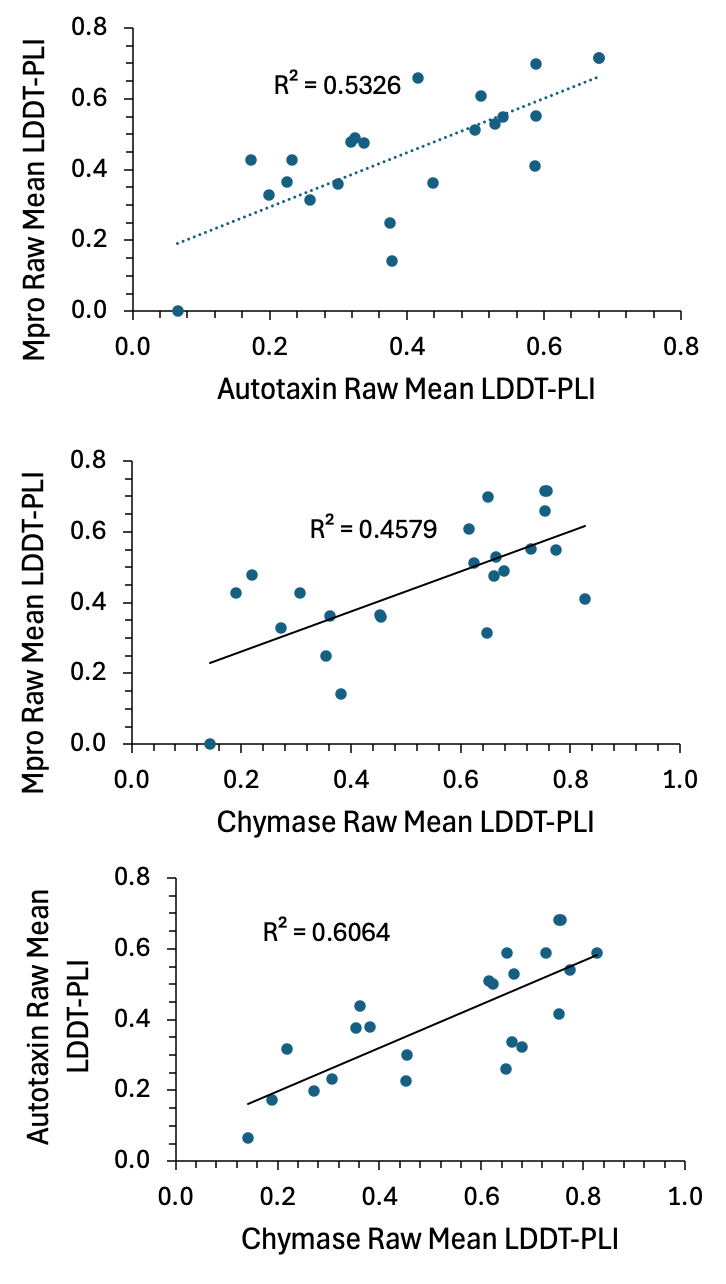 |
| --- |
| Figure S5. Scatter plots of mean accuracy (LDDT-PLI) of groups for pairs of supertargets, computed without penalizing skipped predictions. Each dot represents one group. A group was included here only if it contained predictions for at least 17 chymase predictions, 167 autotaxin predictions, and 15 Mpro predictions. Cathepsin G and WDR55, with only 2 and 1 targets apiece, are omitted here. |

| 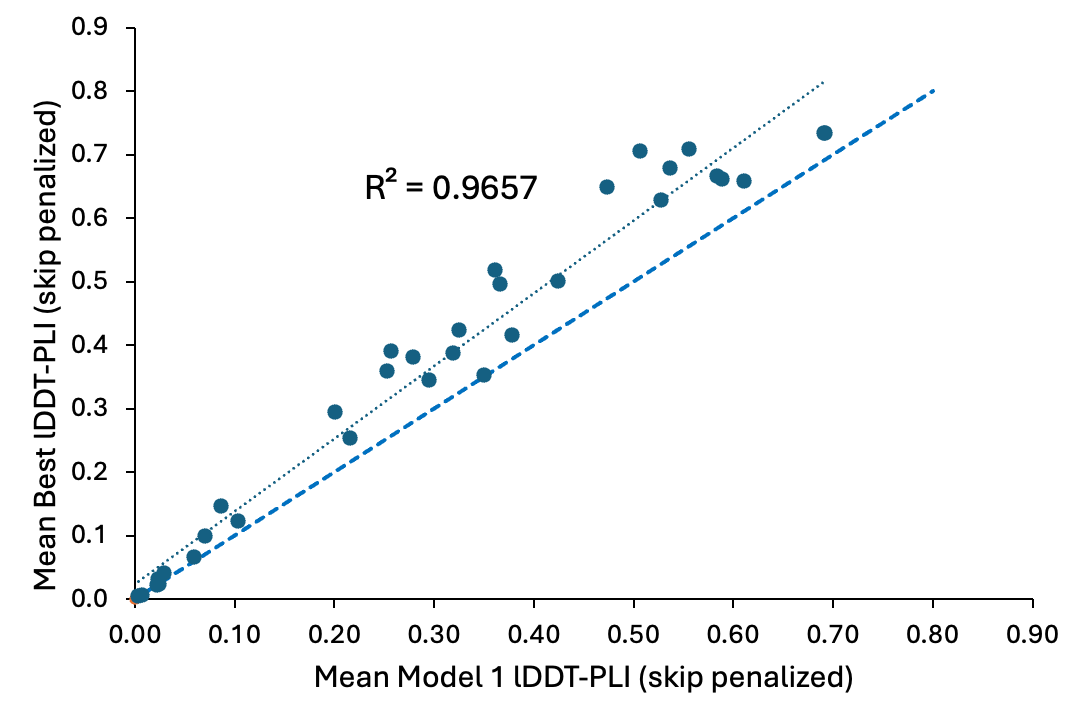 |
| --- |
| Figure S6. Scatter plot of the mean best LDDT-PLI across models vs the mean Model 1 LDDT-PLI. Each dot is one group. |

| 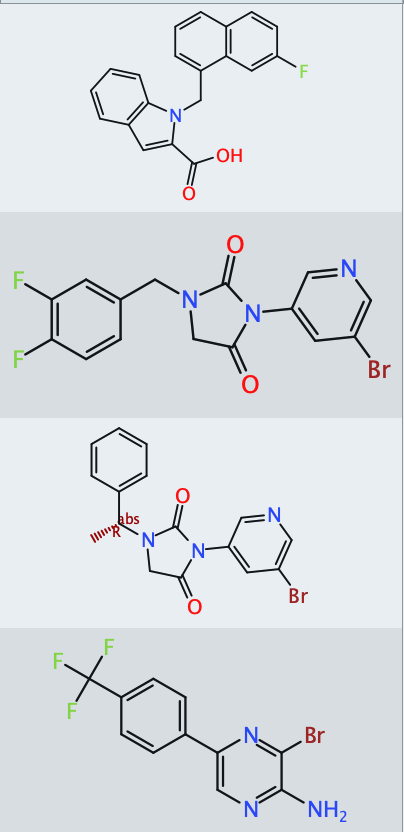 | 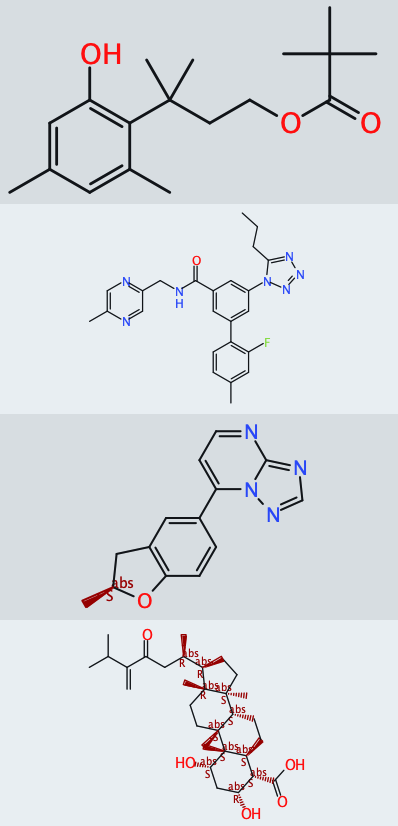 |
| --- | --- |
| Figure S7. Four ligands with the highest (0.70-0.73) and lowest (0.24-0.31) mean best LDDT-PLI across all submitted models, without skip penalty, averaged across groups. The target IDs of the high-accuracy ligands are 1010, 4028, 4027, and 3192. The target IDs of the low-accuracy ligands are 3083, 3058, 5001, and 3109. | |

| 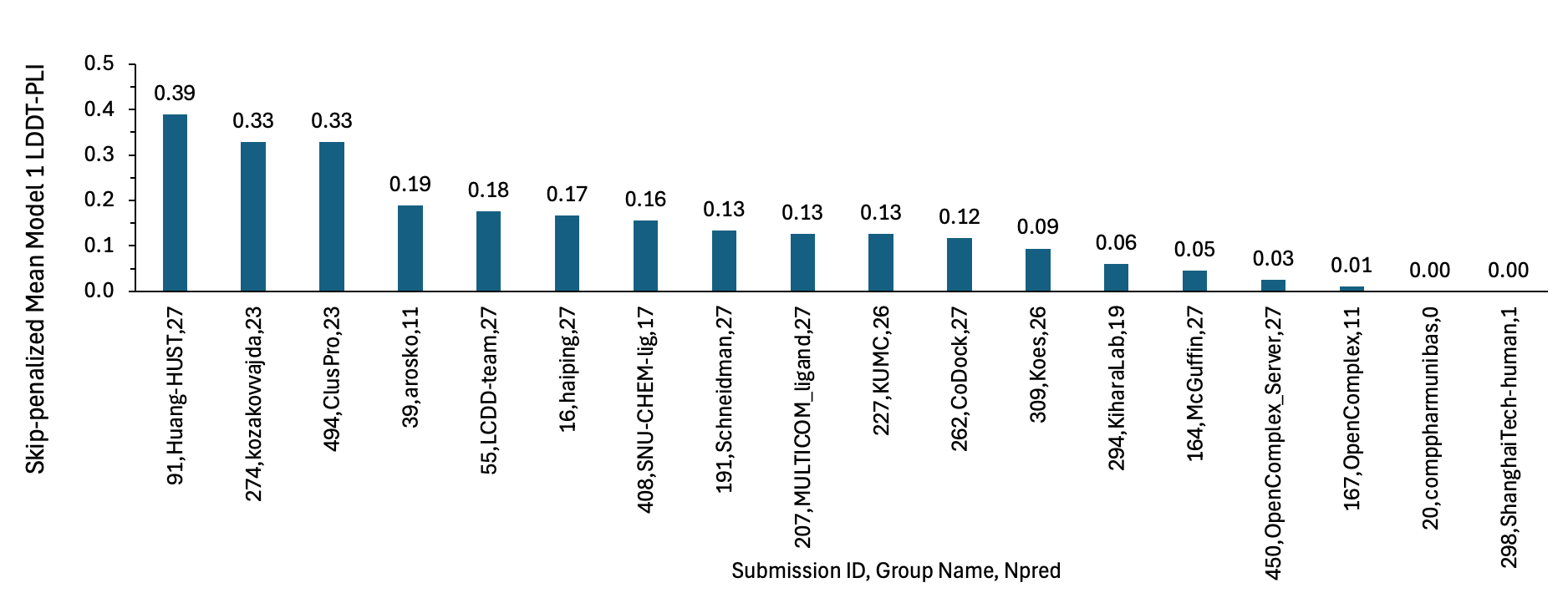 |
| --- |
| Figure S8. Pose prediction accuracy, by group, for the incidental (non-drug-like) ligands. |

| 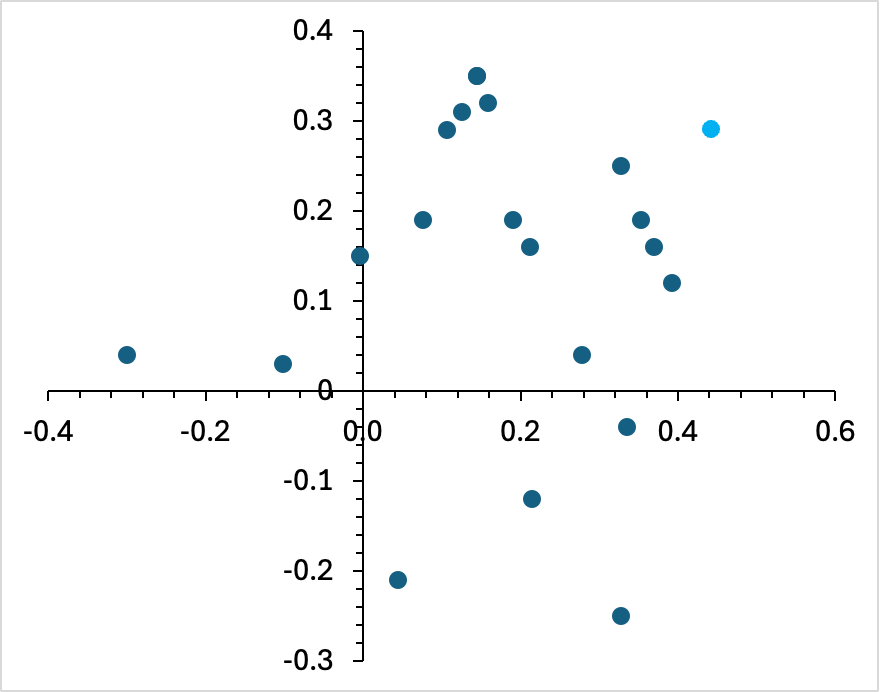 |
| --- |
| Figure S9. Scatter plot of Kendall’s 𝛕 for chymase (vertical axis) vs autotaxin (horizontal), computed without any skip penalty. Each point represents one group. The pale blue point at the top right corresponds to group 16. |
